# Manganese acting as a high-performance heterogeneous electrocatalyst in carbon dioxide reduction

**DOI:** 10.1038/s41467-019-10854-1

**Published:** 2019-07-05

**Authors:** Bingxing Zhang, Jianling Zhang, Jinbiao Shi, Dongxing Tan, Lifei Liu, Fanyu Zhang, Cheng Lu, Zhuizhui Su, Xiuniang Tan, Xiuyan Cheng, Buxing Han, Lirong Zheng, Jing Zhang

**Affiliations:** 1 Beijing National Laboratory for Molecular Sciences (BNLMS), CAS Key Laboratory of Colloid, Interface and Chemical Thermodynamics, CAS Research/Education Center for Excellence in Molecular Sciences, Institute of Chemistry, Chinese Academy of Sciences, Beijing, 100190 P. R. China; 2 School of Chemical Sciences, University of Chinese Academy of Sciences, Beijing, 100049 P. R. China; 3 Beijing Synchrotron Radiation Facility (BSRF), Institute of High Energy Physics, Chinese Academy of Sciences, Beijing, 100049 P. R. China

**Keywords:** Catalyst synthesis, Electrocatalysis, Nanoscale materials

## Abstract

Developing highly efficient electrocatalysts based on cheap and earth-abundant metals for CO_2_ reduction is of great importance. Here we demonstrate that the electrocatalytic activity of manganese-based heterogeneous catalyst can be significantly improved through halogen and nitrogen dual-coordination to modulate the electronic structure of manganese atom. Such an electrocatalyst for CO_2_ reduction exhibits a maximum CO faradaic efficiency of 97% and high current density of ~10 mA cm^−2^ at a low overpotential of 0.49 V. Moreover, the turnover frequency can reach 38347 h^−1^ at overpotential of 0.49 V, which is the highest among the reported heterogeneous electrocatalysts for CO_2_ reduction. In situ X-ray absorption experiment and density-functional theory calculation reveal the modified electronic structure of the active manganese site, on which the free energy barrier for intermediate formation is greatly reduced, thus resulting in a great improvement of CO_2_ reduction performance.

## Abstract (web summary)

While renewable CO_2_ conversion provides a means to remove and recycle waste emissions, there are few earth-abundant materials that are both efficient and active. Here, authors prepare N-doped carbon with atomic manganese as high-performance CO_2_ reduction electrocatalyst.

## Introduction

Electrochemical carbon dioxide (CO_2_) reduction reaction (CO_2_RR) in aqueous solution is a promising approach for both decreasing atmospheric CO_2_ concentration and producing useful fuels^1–3^. How to overcome the limitations of the activation of CO_2_ into CO_2_^•−^ radical anion or other intermediates is the critical bottleneck^4^, especially at low applied overpotentials in terms of avoiding hydrogen evolution and reducing energy consumption. The heterogeneous electrocatalysts based on noble metals Pd, Ag, Au and their alloys have shown to be active for CO_2_RR in aqueous solution at low overpotentials^5–11^. It is desirable to develop efficient and selective heterogeneous electrocatalysts for CO_2_RR based on cheap and earth-abundant metals. Transition metal manganese (Mn) is the third after iron and titanium among the transition elements in its natural abundance. However, the studies on Mn-based heterogeneous electrocatalysts for CO_2_RR have been rarely reported. Strasser and coworkers synthesized a family of metal-nitrogen-doped carbon (M-N-C) electrocatalysts including a variety of transition metals such as Mn, Fe, Co, Ni, and Cu for CO_2_ reduction^12,13^. The electrocatalytic performance of Mn-N-C remains largely limited, especially as compared with Fe-N-C and Ni-N-C catalysts^13–15^. It is urgent to develop the efficient Mn-based heterogeneous electrocatalysts with high CO_2_RR activity and selectivity, but is challenging.

Herein, we show that the Mn-based heterogeneous catalyst can be efficient and selective electrocatalyst for converting CO_2_ to carbon monoxide (CO) via halogen and nitrogen dual-coordination to modulate the electronic structure of Mn atoms. Outstandingly, the CO faradaic efficiency (FE_CO_) is up to 97% with a current density of ~10 mA cm^−2^ at a low overpotential of 0.49 V. Moreover, the turnover frequency (TOF) for CO_2_RR can reach 38347 h^−1^ at overpotential of 0.49 V, which outperforms all the reported heterogeneous electrocatalysts under similar conditions. The in situ synchrotron X-ray absorption spectra (XAS) for CO_2_RR and theoretical simulations reveal the promoted CO_2_ activation process and facilitated CO desorption process. This study opens up an opportunity for improving the CO_2_RR properties of metal electrocatalysts under mild conditions, particularly for the metals with intrinsically low activity.

## Results

### Synthesis and structural characterizations of (Cl, N)-Mn/G

**Fig. 1:**
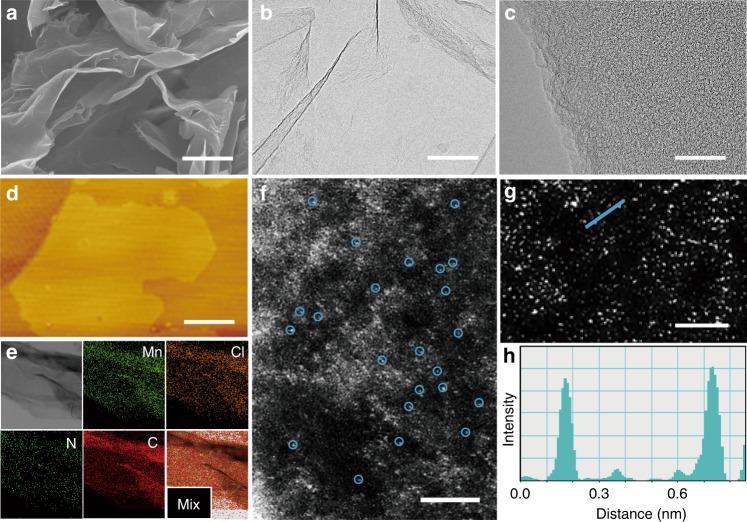
Structural characterizations of (Cl, N)-Mn/G. **a** SEM image. **b**, **c** TEM images. **d** AFM image. **e** EDS image. **f** HAADF-STEM image. The high density bright dots (highlighted by blue circles) corresponding to single Mn atoms are homogeneously distributed across the entire carbon framework. **g** Enlarged HAADF-STEM image of Fig. 1f. **h** The corresponding intensity profile along the line as shown in Fig. 1g. Scale bars, 1.5 µm in (**a**), 200 nm in (**b**), 50 nm in (**c**), 800 nm in (**d**), 2 nm in (**f**) and 1 nm in (**g**)

The Mn-based heterogeneous catalyst was synthesized by pyrolyzing a pre-formed crystalline polymer Mn-EDA-Cl (EDA = ethylenediamine) (Supplementary Figs. 1–3). The X-ray diffraction (XRD), scanning and transmission electron microscopes (SEM, TEM), and atomic force microscopy (AFM) images show the formation of graphitic carbon layer, with a lateral size of ~3 µm and thickness of ~1.1 nm (Fig. 1a–d, Supplementary Figs. 4, 5). No particle decoration was observed on graphene (G) from TEM images, which is consistent with the XRD results that the diffractions from metallic Mn are absent (Supplementary Fig. 4). Energy-dispersive X-ray spectroscopy (EDS) images reveal that Mn, Cl and N elements homogeneously distribute over the entire graphene (Fig. 1e). The aberration-corrected high-angle annular dark-field scanning transmission electron microscopy (HAADF-STEM) images confirm the atomically dispersed Mn on graphene (Fig. 1f–h). The Mn content is 0.049 wt% measured by inductively coupled plasma-optical emission spectrometer analysis (Supplementary Table 1). The as-synthesized electrocatalyst was denoted as (Cl, N)-Mn/G.

### Fine structures of (Cl, N)-Mn/G

**Fig. 2:**
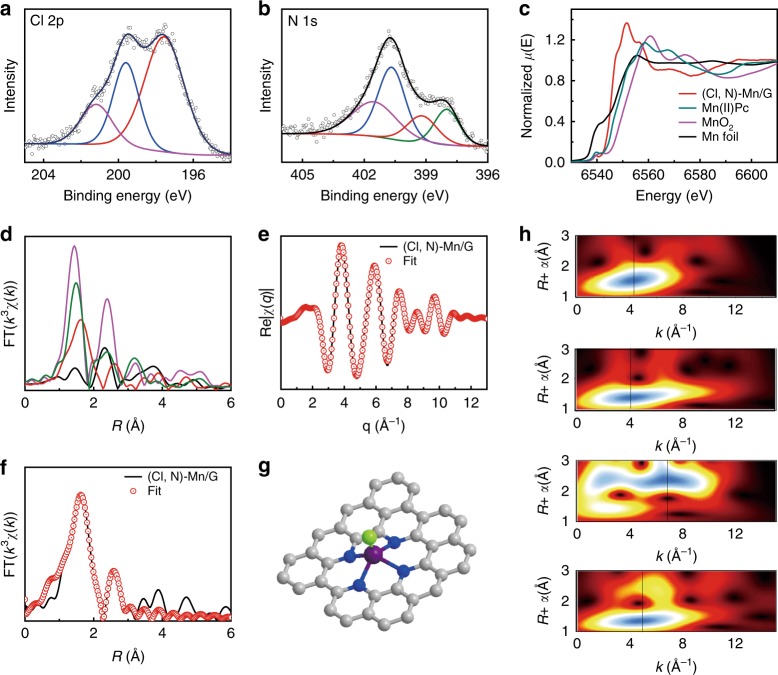
Fine structure of (Cl, N)-Mn/G. **a**, **b** High-resolution Cl 2p and N 1s XPS spectra. **c**, **d** XANES and EXAFS spectra at Mn K-edge. (**e**) EXAFS fitting curves of (Cl, N)-Mn/G in *q* space. **f** EXAFS fitting curves of the (Cl, N)-Mn/G in *R* space. **g** Schematic model of (Cl, N)-Mn/G: Mn (purple), Cl (green), N (blue), and C (gray). **h** WT of (Cl, N)-Mn/G, Mn(II)Pc, Mn foil and MnO_2_ (from top to bottom)

The insightful chemical and structural information of the (Cl, N)-Mn/G catalyst was examined by X-ray photoelectron spectra (XPS). The full XPS survey scan proves the presence of Mn, Cl, N and C in the catalyst (Supplementary Fig. 6). The high-resolution Cl 2p XPS spectrum (Fig. 2a) shows the peak at 197.7 eV corresponding to Mn-Cl species^16,17^ and peaks at 199.6 eV (2p_3/2_) and 201.2 eV (2p_1/2_) corresponding to C-Cl species. The N 1s XPS spectrum (Fig. 2b) reveals four N species, including Mn-N species at 399.2 eV, pyridinic N species at 398.0 eV, pyrrolic N species at 400.7 eV and graphitic N species at 401.5 eV^13,18,19^. The XPS results indicate that Cl and N co-coordinate with Mn atoms. The local structure of the catalyst at atomic level was further investigated by synchrotron XAS. For the Mn K-edge X-ray absorption near-edge structure (XANES, Fig. 2c), the (Cl, N)-Mn/G catalyst exhibits similar spectrum features to that of reference sample Mn(II) phthalocyanine (Mn(II)Pc), suggesting that (Cl, N)-Mn/G possesses comparable *D*_4h_ symmetry as Mn(II)Pc^20,21^. The absorption edge position of (Cl, N)-Mn/G is located between those of Mn foil and Mn(II)Pc, implying the valence of Mn species in (Cl, N)-Mn/G is less than +2. The phase-uncorrected Fourier-transformed (FT) extended X-ray absorption fine structure (EXAFS) of (Cl, N)-Mn/G presents the main peak at 1.63 Å (Fig. 2d), corresponding to the scattering interaction between Mn atoms and Cl/N in the first shell of (Cl, N)-Mn/G. Compared to the EXAFS spectrum of the reference Mn(II)Pc, which shows a symmetric peak (Mn-N_4_) with Mn-N scattering path at 1.49 Å, (Cl, N)-Mn/G presents an unsymmetric peak with slight migration. This can be attributed to the additional coordination of Mn-Cl in (Cl, N)-Mn/G and the caused distortion of Mn atoms out of graphene plane. It is worth noting that the peak from Mn-Mn scattering path (~2.3 Å)^22,23^ was not observed for (Cl, N)-Mn/G, further proving the presence of atomically dispersed Mn. The peak at around 2.60 Å can be attributed to the Mn-C scattering path in higher shells^22–24^. The coordination configuration of Mn atom for (Cl, N)-Mn/G was further investigated by quantitative EXAFS curve fitting analysis (Fig. 2e, f, Supplementary Fig. 7 and Table 2)^20,25–28^. The best-fitting analyses clearly confirm that Mn centre is coordinated with one Cl atom and four N atoms, as illustrated in Fig. 2g. The calculated Mn-Cl mean bond distance is 2.39 Å and Mn-N mean bond distance is 2.08 Å in (Cl, N)-Mn/G, which is longer than that in Mn(II)Pc (1.95 Å) with the ideal square planar symmetry. The wavelet transform (WT) results give further support for the existence of Mn-Cl/N bonding (with a maximum at 4.3 Å^−1^) in the (Cl, N)-Mn/G catalyst, as compared with Mn(II)Pc, Mn foil and MnO_2_ (Fig. 2h). Moreover, the soft XAS of N K-edge and C K-edge further confirms that the single Mn site bonds to N and excludes the existence of Mn-C bond in (Cl, N)-Mn/G catalyst (Supplementary Fig. 8)^23,29^.

### Electrochemical activities of CO_2_ reduction

**Fig. 3:**
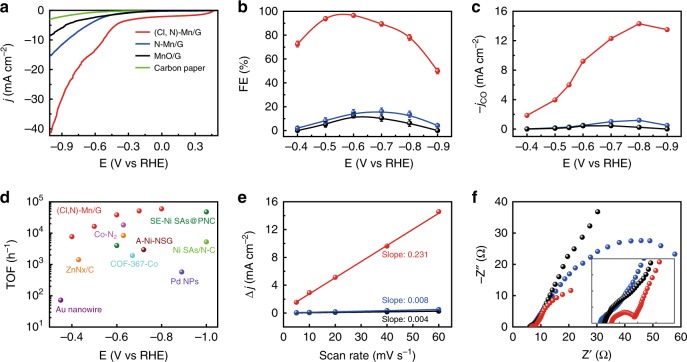
Electrochemical CO_2_RR performance on (Cl, N)-Mn/G catalyst and control samples of N-Mn/G, MnO/G and carbon paper. **a** LSV curves of different catalysts (in a CO_2_-saturated 0.5 M KHCO_3_ solution, scanning rate: 10 mV s^−1^). **b** FE_CO_ at various applied potentials. **c** Potential-dependent CO partial current density. **d** TOF of (Cl, N)-Mn/G catalyst compared with those of other CO_2_ to CO reduction catalysts in the Supplementary Table 5^7–13,29,31-41^. **e** Charging current density differences plotted against scan rates. **f** EIS spectra. The inset shows the enlarged Nyquist plots for high frequency region. All data in b are presented as mean ± s.d.

The CO_2_ electrolysis of the (Cl, N)-Mn/G catalyst was determined by linear sweep voltammetry (LSV) in a CO_2_-saturated 0.5 M KHCO_3_ solution. For comparison, the N-coordinated Mn single atoms supported by graphene (N-Mn/G) and MnO nanoparticles supported by graphene (MnO/G) were also synthesized and measured (Supplementary Figs. 9–12). For (Cl, N)-Mn/G catalyst, a cathodic peak occurs at −0.6 V (vs RHE) in LSV curve (Fig. 3a and Supplementary Fig. 13) and the peak current increases linearly with scan rate (Supplementary Fig. 14), which suggests that this peak originates from CO_2_ mass transfer controlled electrolysis process^20,30^. Such a cathodic peak was not observed for the other two reference catalysts (Fig. 3a and Supplementary Fig. 13). Moreover, the (Cl, N)-Mn/G catalyst shows the absence of the cathodic peak and much low current density in N_2_-saturated KHCO_3_ solution (Supplementary Fig. 13), indicating that the (Cl, N)-Mn/G is indeed active for catalyzing CO_2_RR. The (Cl, N)-Mn/G catalyst shows current densities of 13.4 mA cm^−2^ (normalized by the geometrical surface area) at the potential of −0.6 V (*vs* RHE), which is 3.7-fold and 5.8-fold relative to N-Mn/G (3.6 mA cm^−2^) and MnO/G (2.3 mA cm^−2^), respectively. The electrolysis test was performed in an H-type electrochemical cell separated by a Nafion 117 membrane and the products were analyzed by ^1^H nuclear magnetic resonance spectroscopy and gas chromatography. CO and H_2_ were the only products in the gas phase and no liquid products were detected in the potential range from −0.4 to −0.9 V (vs RHE) (Supplementary Fig. 15). The FE_CO_ reaches a maximum of 97% over (Cl, N)-Mn/G catalyst at −0.6 V (vs RHE), which is significantly higher than that of N-Mn/G and MnO/G (<17%) at the full potential range from −0.4 to −0.9 V (vs RHE) (Fig. 3b). By using ^13^CO_2_ as the feedstock for CO_2_RR, it is confirmed that CO is the CO_2_ reduction product (Supplementary Fig. 16). Furthermore, the CO_2_ reductions catalyzed by (Cl, N)-Mn/G in different CO_2_-saturated electrolytes (0.5 M Na_2_SO_4_, KCl, and K_2_SO_4_ solutions) show much enhanced current densities, remarkable cathodic peaks in LSV curves and high FE_CO_ values up to 95% (Supplementary Fig. 17), further proving that it is CO_2_ to be really reduced. In addition, the CO partial current density of (Cl, N)-Mn/G was plotted against the applied potential. The (Cl, N)-Mn/G shows a maximum value of *j*_CO_ = 14.3 mA cm^−2^ at −0.8 V (vs RHE), whereas those of the two reference electrocatalysts are <1.3 mA cm^−2^ (Fig. 3c). Moreover, the N doped graphene (in absence of Mn) was synthesized and tested for CO_2_RR, which shows a very low activity (1.2 mA cm^−2^ at −0.6 V (vs RHE) and FE of 9.5%, Supplementary Fig. 18). It confirms that the high current density and FE_CO_ of (Cl, N)-Mn/G in CO_2_RR originates from the coordinated Mn in the carbon framework. To further verify this, SCN^−^, a commonly adopted ion to poison metal sites, was used as an indicator for active sites^29,31^. There is a remarkable depression of catalytic activity for (Cl, N)-Mn/G by blocking of Mn atoms with SCN^-^, confirming the active site role of the coordinated Mn structure (Supplementary Fig. 19). Outstandingly, as shown in Fig. 3d, the (Cl, N)-Mn/G catalyst displays a high TOF value of 38347 h^−1^ for CO_2_RR at −0.6 V (*vs* RHE), which is much higher than that of N-Mn/G (744 h^−1^) (Supplementary Fig. 20) and outperforms all the reported heterogeneous electrocatalysts under similar conditions^7–13,29,31–41^ (Supplementary Fig. 21 and Table 5). According to the measured double-layer capacitance, in which the slope could be a reference of electrochemical active surface area (ECSA)^42^ (Fig. 3e and Supplementary Fig. 22), the ECSA for (Cl, N)-Mn/G is roughly 29 and 58 times higher than those of N-Mn/G and MnO/G, respectively. It suggests that the (Cl, N)-Mn/G catalyst can afford more accessible surface in electrolytic process, partially contributing to its enhanced activity^42^. The specific surface areas of (Cl, N)-Mn/G, N-Mn/G and MnO/G were determined to be 1192, 425 and 156 m^2^ g^−1^, respectively (Supplementary Fig. 23). The electrochemical performance gap is much higher than the specific surface area gap between the three catalysts, suggesting the enhanced inherent activity of (Cl, N)-Mn/G^43^. Electrochemical impedance spectroscopy (EIS) was carried out to gain further insight into CO_2_RR kinetics. The Nyquist plots demonstrate that (Cl, N)-Mn/G shows much smaller interfacial charge-transfer resistance during CO_2_ reduction process (Fig. 3f), suggesting a favorable faradaic process. The CO_2_ reduction test for 12 h at −0.6 V (vs RHE) demonstrates the relative stability of (Cl, N)-Mn/G catalyst (Supplementary Fig. 24).

### In situ XAS spectra of (Cl, N)-Mn/G in electrochemical CO_2_RR

**Fig. 4:**
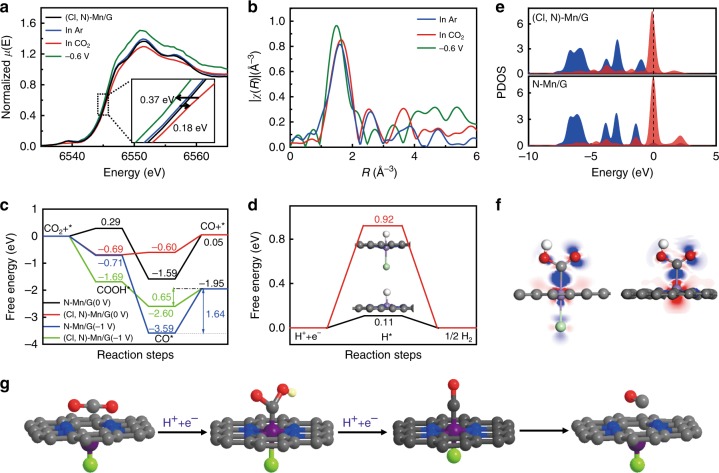
In situ XAS experiment and DFT calculation. **a** Normalized XANES of (Cl, N)-Mn/G catalyst under various conditions (inset is the magnified image). **b** Fourier transform magnitudes of EXAFS spectra of (Cl, N)-Mn/G. **c** Calculated free energy of CO_2_RR. **d** Calculated free energy of hydrogen adsorption. **e** Projected density of states (PDOS) of the COOH* 2p state (blue-shaded areas) and d-projected DOS of Mn (red-shaded areas) in the adsorption structures for (Cl, N)-Mn/G and N-Mn/G, respectively. **f** Electron density difference for COOH* adsorbed on (Cl, N)-Mn/G (left) and N-Mn/G (right). The blue and red denote the electron accumulation and electron depletion, respectively. **g** Structural evolution of the active site for (Cl, N)-Mn/G in electrochemical CO_2_RR (Mn: purple, Cl: green, N: blue, O: red, H: white and C: gray)

To elucidate the structure and chemical state of the active sites of (Cl, N)-Mn/G catalyst during CO_2_RR, the in situ XAS spectra were recorded under operando conditions at catalytic state. The XANES shows that the Mn K-edge of as-prepared (Cl, N)-Mn/G is shifted to higher energy after immersing in CO_2_ saturated 0.5 M KHCO_3_ (Fig. 4a), which can be attributed to the increased oxidation of the Mn sites due to the charge transfer from low-valence Mn to the carbon 2p orbital in CO_2_ to form a CO_2_^δ−^ species^44,45^. During electrochemical CO_2_ reduction at −0.6 V (vs RHE), the Mn K-edge shifts back to lower energy that is even lower than that of the original (Cl, N)-Mn/G catalyst. It indicates the ongoing CO_2_ reduction and high activation ability of Mn center for CO_2_^45^. However, the in situ Mn K-edge XANES spectra of N-Mn/G show little changes under similar operating conditions (Supplementary Fig. 25), suggesting the important role of Cl coordination to the oxidation states of Mn in CO_2_RR process. Fourier transform of the EXAFS shows that the intensity of the main peak at approximately 1.62 Å slightly increases (Fig. 4b), which can be ascribed to the additional Mn-C bond^46^ from the CO_2_ interacted with Mn center. The main peak is shifted to lower length (∼0.14 Å) and the Mn-Cl bond is enlarged during CO_2_ reduction at −0.6 V (vs RHE), which suggests the recovery of distortion of Mn atoms to form a stable plane structure due to the additional Cl interaction^47^.

### Density functional theory (DFT) calculations and electrochemical CO_2_RR mechanism

DFT calculations were performed to further understand the intrinsic property and reactivity of (Cl, N)-Mn/G catalyst. The formation of adsorbed intermediate COOH* was investigated for the potential limiting step, which is the initial reduction barrier for CO_2_-to-CO reduction^10,11,48^^,^. The optimized configurations of (Cl, N)-Mn/G show the distortion of Mn atoms out of graphene plane (Supplementary Fig. 26a), which would promote the adsorption of CO_2_ and COOH* intermediates on catalyst surface^49^ (Supplementary Fig. 27). After interacting with COOH* intermediates (Supplementary Fig. 26b and Table 4), the (Cl, N)-Mn/G presents a recovered in-plane structure of Mn atom and the Mn-Cl bond length (2.31 Å) is longer than that of the original (Cl, N)-Mn/G (2.17 Å), which is in accord with the in situ XAS experiment results. By contrast, N-Mn/G shows a distorted structure of Mn center after adsorbing intermediate COOH*, presenting lower adsorption energy (*∆E*_ads_ = −1.62 eV) than that of (Cl, N)-Mn/G (*∆E*_ads_ = −2.36 eV) (Supplementary Fig. 26c, d and Table 3). The free energy profiles of CO_2_RR catalyzed by (Cl, N)-Mn/G and N-Mn/G are shown in Fig. 4c and Supplementary Fig. 28. Compared with N-Mn/G, there are an improvement of *Δ*G for the first step (0.98 eV) and a decrease of CO desorption free energy (0.99 eV) for (Cl, N)-Mn/G. As a result, the CO_2_RR on (Cl, N)-Mn/G is much more effective than that on N-Mn/G. At the potential of *U* = −1.0 V vs RHE, the negative potential further favors the formation of COOH*. CO release process can also be optimized on (Cl, N)-Mn/G, according to the much lower energy for CO* desorption (0.65 eV) than that of N-Mn/G (1.64 eV). Moreover, the free energy profiles of hydrogen adsorption on the two catalysts were calculated. The (Cl, N)-Mn/G shows much higher formation energy for H* adsorption (0.92 eV) than that of N-Mn/G (0.11 eV), proving that the H_2_ evolution reaction is well inhibited on (Cl, N)-Mn/G (Fig. 4d and Supplementary Fig. 29).

The electronic structures of the Mn atoms with different ligand conditions were further calculated. The position of d-band center (*E*_dbc_) for (Cl, N)-Mn/G shows a downshift of 0.38 eV after interacting with COOH* (Fig. 4e, Supplementary Fig. 30 and Table 4), which is much larger than that in N-Mn/G (0.12 eV). It suggests more electrons transferred between COOH* and (Cl, N)-Mn/G owing to the contribution of Cl coordination (Supplementary Fig. 31)^20,50^. Accordingly, the COOH* on (Cl, N)-Mn/G shows wider PDOS than that on N-Mn/G due to the more gain electrons on the former. The additional electrons transfered between Mn and Cl in (Cl, N)-Mn/G can also be observed in Fig. 4f. Similarly, there exists significant electron transfer in Mn-C bonding area between COOH* and (Cl, N)-Mn/G, suggesting a strong interaction between these two parts. Based on above analyses, the structural evolution of the active site during electrochemical CO_2_RR is proposed (Fig. 4g). The Cl coordination induces distorted single-atom Mn center to facilitate the adsorption of CO_2_ and COOH*, and then stabilizes a low-energy transition state as well as facilitates the final CO desorption process. These processes are in favor of reducing the energy barrier of intermediate formation for electrochemical CO_2_RR.

## Discussion

The above results prove that the electronic state of the single Mn active center could be tuned by changing its adjacent chemical environment during electrochemical process through Cl and N dual-coordination. The performing principle for high-performance electrocatalytic CO_2_RR was expanded to other systems. First, the (Br, N)-Mn/G and (I, N)-Mn/G catalysts were synthesized by the similar route for synthesizing (Cl, N)-Mn/G catalyst, which exhibit excellent activities and selectivities for electrocatalytic CO_2_RR (Supplementary Figs. 32–35). For example, (Br, N)-Mn/G and (I, N)-Mn/G catalysts respectively show FE_CO_ of 92% at −0.6 V (vs RHE) and 89% at −0.5 V (vs RHE), which is 6.4-fold and 10.4-fold FE_CO_ of halogen-free N-Mn/G, respectively (Supplementary Fig. 36). Second, the (Cl, N)-Fe/G and (Cl, N)-Co/G were synthesized and both of them exhibit much enhanced performance for electrocatalytic CO_2_RR compared to their counterparts without Cl coordination (Supplementary Figs. 37 and 38). The electrocatalytic activities of (Cl, N)-Fe/G and (Cl, N)-Co/G are lower than that of (Cl, N)-Mn/G. In other words, the positive effect of Cl is more dramatic when the metal is Mn as compared with the effect for Co and Fe, indicating that the performance of (Cl, N)-M/G catalyst has a strong dependence on the nature of transition metal.

In summary, we demonstrate that the electrocatalytic activity of Mn-based heterogeneous catalyst can be significantly improved through halogen and nitrogen dual-coordination to modulate the electronic structure of Mn atom. The maximum FE_CO_ can reach 97% with a high current density of ~10 mA cm^−2^ at a low overpotential of 0.49 V. Moreover, the TOF value for CO_2_RR is up to 38347 h^−1^ at overpotential of 0.49 V, which outperforms all the reported heterogeneous electrocatalysts including noble metal catalysts. The superior catalytic performance originates from the modified electronic structure of the active Mn site, on which the free energy barrier for rate-limiting step of the intermediate formation is greatly reduced. This work provides a promising method for improving the CO_2_RR properties of metal electrocatalysts under mild conditions, especially for the metals with intrinsically low activity. We anticipate that the highly efficient and low-cost Mn-based heterogeneous catalyst may find more applications in other electrochemical reactions.

## Methods

### Materials

Anhydrous manganese chloride (99.99%), cobalt nitrate hexahydrate (>99%), cobalt chloride hexahydrate (99.9%), potassium bicarbonate (99%), hydrochloric acid (grade), ethanol (A. R. grade) and urea (99%) were provided by Sinopharm Chemical Reagent Co., Ltd. Manganese bromide hydrate (98%), manganese nitrate tetrahydrate (>98%), Iron chloride (99%), Iron nitrate nonahydrate (99%), ethylenediamine (EDA) (99.5%), potassium iodide (99%), citric acid (99.5%), Nafion D-521 dispersion (5% w/w in water and 1-propanol, ≥0.92 meg/g exchange capacity), Nafion N-117 membrane (0.180 mm thick, ≥0.90 meg/g exchange capacity) and Toray Carbon Paper (CP, TGP-H-60, 19 × 19 cm) were purchased from Alfa Aesar China Co., Ltd. CO_2_ (>99.999%) was provided by Beijing Analysis Instrument Factory.

### Synthesis of (Cl, N)-Mn/G and N-Mn/G

Typically, anhydrous manganese chloride (1.0 g) was dissolved in 30 mL of ethanol. Then 1 mL ethylenediamine was added to the above solution under vigorous stirring for 10 h at room temperature. After the reaction, the product was separated by centrifugation and washed with ethanol, and finally dried overnight at 60 °C. The obtained precursor was annealed at 900 °C for 1 h in nitrogen atmosphere with a ramping rate of 3 ^o^C min^−1^. After cooling down to room temperature, the (Cl, N)-Mn/G was obtained by leaching the calcined product with HCl to remove the free standing metallic residues. The N-Mn/G was obtained under the same procedure except that the manganese chloride was replaced by manganous nitrate

### Synthesis of MnO/G and N-G

In a typical synthesis, urea (3.0 g), citric acid (1.0 g) and manganous nitrate (0.5 g) were dispersed in deionized water (5 mL) under continuous stirring for 0.5 h and then dried at 80 °C. The obtained solid was annealed at 900 °C for 1 h at a heating rate of 3 °C min^−1^ under nitrogen flow. After cooling down to room temperature, the final powder sample was collected. The N-G sample was synthesized by a route similar to that for MnO/G preparation, involving no manganous nitrate.

### Synthesis of (Br, N)-Mn/G and (I, N)-Mn/G

For the synthesis of (Br, N)-Mn/G, manganese bromide hydrate (2.27 g) was firstly dissolved in 30 mL of ethanol. Then 1 mL ethylenediamine was added to the above solution under vigorous stirring for 10 h at room temperature. After reaction, the product was separated by centrifugation and washed with ethanol, and finally dried overnight at 60 °C. The obtained precursor was annealed and processed under same procedure for (Cl, N)-Mn/G synthesis to obtain the (Br, N)-Mn/G. The (I, N)-Mn/G was synthesized under the same procedure except that manganese bromide was replaced by manganese nitrate tetrahydrate (2.0 g) and potassium iodide (1.0 g).

### Synthesis of (Cl, N)-Fe/G and N-Fe/G

(Cl, N)-Fe/G and N-Fe/G were obtained by the route for synthesizing (Cl, N)-Mn/G and N-Mn/G under the same procedure except that manganese salt was replaced by cobalt chloride and cobalt nitrate, respectively.

### Synthesis of (Cl, N)-Co/G and N-Co/G

(Cl, N)-Co/G and N-Co/G were obtained by the route for synthesizing (Cl, N)-Mn/G and N-Mn/G under the same procedure except that manganese salt was replaced by cobalt chloride and cobalt nitrate, respectively.

### Characterizations

Powder X-ray diffraction pattern was performed on a Rigaku D/max-2500 diffractometer with Cu Kα radiation (*λ* = 1.5418 Å) at 40 kV and 200 mA. The morphologies were characterized by SEM (HITACHI S-4800), TEM (JEOL-1010) operated at 100 kV and HRTEM (JEOL-2100F) operated at 200 kV. The high-angle annular dark-field scanning transmission electron microscopy (HAADF-STEM) characterization was performed on a JEOL JEM-ARF200F TEM/STEM with a spherical aberration corrector. AFM measurement was performed on a tapping-mode atomic force microscope (Nanoscope IIIa, Digital Instruments, Santa Barbara, CA), with a silicon cantilever probes. XPS was determined by VG Scientific ESCALab220i-XL spectrometer using Al Kα radiation. The 500 μm X-ray spot was used. The base pressure in the analysis chamber was about 3 × 10^–10^ mbar. The element contents of Mn were determined by ICP-AES (VISTA-MPX). The elemental contents of C, Cl and N were determined using Flash EA1112 from Thermo. Data of XAFS were processed using the Athena and Artemis programs of thee IFEFFIT package based on FEFF 6. Prior to merging, the spectra were aligned to the first and largest peak in the smoothed first derivative of the absorption spectrum, background removed, and normalized. Data were processed with *k*^2^-weighting and an Rbkg value of 1.0. Merged data sets were aligned to the largest peak in the first derivative of the adsorption spectrum. Normalized μ(E) data were obtained directly from the Athena program of the IFEFFIT package. The quantitative structural parameters were obtained via a least-squares curve parameter fitting method using ARTEMIS module.

### Electrochemical test

The catalysts (1.0 mg) and 10 μL of a 5 wt% Nafion solution were ultrasonically mixed with 100 µL of ethanol to form inks. A loading of 0.5 mg cm^−2^ was obtained on the surface of the CP electrode by dropping the catalyst ink. Electrocatalytic CO_2_RR was evaluated in a H-type electrochemical cell with three electrode system in CO_2_-saturated 0.5 M KHCO_3_ electrolyte. A Pt gauze and an Ag/AgCl (3.5 M KCl) were used as the counter electrode and reference electrode, respectively. The working and reference electrodes were placed in the cathode chamber, while the counter electrode was placed in the anode chamber, which was separated by a piece of Nafion 117 ionic exchange membrane to avoid the re-oxidation of CO_2_RR-generated products. The electrolyte was bubbled with N_2_ or CO_2_ for at least 30 min to form N_2_ or CO_2_ saturated solution and maintained this flow rate during measurements. LSV test was performed in CO_2_-saturated 0.5 M KHCO_3_ solution with a scan rate of 10 mV/s. ECSA referred the CV results under the potential windows of 0.23 V ~ 0.13 V (vs RHE). EIS measurements were carried out by applying at −0.5 V (vs RHE) with 5 mV amplitude in a frequency range from 100 KHz to 100 mHz. For the faradaic efficiency analysis, gas products were detected by gas chromatograph (GC, HP 4890D), which was equipped with FID and TCD detectors using helium as the internal standard. The liquid product was analyzed by ^1^H NMR on Bruker AVANCE AV III 400. The isotope-labeled experiment was performed using ^13^CO_2_ and the gas products were analyzed using gas chromatography-mass spectrometry (GC-MS, 7890A and 5975C, Agilent).

### Evaluation of TOF

The TOF for CO was calculated as follows:

1 $${\mathrm{TOF}}\;\left( {{\mathrm{h}}^{ - {\mathrm{1}}}} \right) = \frac{{j_{{\mathrm{co}}}/\left( {NF} \right)}}{{m_{{\mathrm{cat}}} \times \omega /M_{{\mathrm{Mn}}}}} \times 3600$$

*j*_CO_: partial current (A) for CO product;

*N*: the number of electron transferred for product formation, which is 2 for CO;

*F*: faradaic constant, 96485 C mol^−1^;

*m*_cat_: catalyst mass in the electrode, g;

*ω*: Mn loading in the catalyst;

*M*_Mn_: atomic mass of Mn, 54.94 g mol^−1^.

### In situ X-ray absorption spectroscopy

The XAFS experiment was carried out at Beamline 1W1B at BSRF. A home-made plastic electrochemical cell was employed for in situ XAS measurement under the sensitive fluorescence model. The cell was filled with electrolyte (0.5 M aqueous KHCO_3_). Ag/AgCl and Pt gauze were used as reference electrode and counter electrode, respectively. The working electrode compartment had walls with a single circular hole of 1.0 cm in diameter. A catalyst/thin carbon paper as the working electrode was in contact with a slip of copper tape and fixed with Kapton (polyimide) tape to the exterior of the wall of the cell, over the 1.0 cm hole, catalyst layer facing inwards. During the measurement, a series of potentials were applied to the working electrode.

### Computational details for calculations

The first principles calculations in the framework of DFT, including structural, electronic performances, were carried out based on the Cambridge Sequential Total Energy Package known as CASTEP. The exchange-correlation functional under the generalized gradient approximation (GGA) with norm-conserving pseudopotentials and Perdew-Burke-Ernzerhof functional was adopted to describe the electron-electron interaction. An energy cutoff of 750 eV was used and a k-point sampling set of 5 × 5 × 1 was tested to be converged. A force tolerance of 0.01 eV Å^−1^, energy tolerance of 5.0 × 10^–7^ eV per atom and maximum displacement of 5.0 × 10^–4^ Å were considered. Each atom in the storage models was allowed to relax to the minimum in the enthalpy without any constraints. The vacuum space along the z direction was set to be 15 Å, which was enough to avoid the interaction between the two neighboring images. The COOH, H, or CO group was adsorbed on the surface of N-Mn/G and (Cl, N)-Mn/G, respectively.

The adsorption energy Δ*E*_ads_ was defined as:

2 $$\Delta E_{\mathrm{ads}} = E_{{\mathrm{after}}}-E_{{\mathrm{before}}} + E_A$$

where *E*_before_ and *E*_after_ denote the total energy of substrates before and after adsorbing A group (COOH, CO, or H), *E*_*A*_ is the energy of A group.

3 $$\Delta G = \Delta E_{\mathrm{ads}} + \Delta E_{{\mathrm{ZPE}}}-T\Delta S$$

where ∆*E* denotes the adsorption energy, ∆*E*_ZPE_ and *∆S* are the changes of zero-point energy and entropy, and the temperature *T* is 300 K.

### Reporting summary

Further information on research design is available in the Nature Research Reporting Summary linked to this article.

## Supplementary information

Supplementary Information

Reporting Summary

## Data Availability

The data supporting the findings of this study are available within the article and its Supplementary information files. All data is available from the authors upon reasonable request.

## Code availability

All code supporting the findings of this study are available from the corresponding author on request.

## References

[1] Turner JA (1999). A realizable renewable energy future. Science.

[2] Rosen BA (2011). Ionic liquid-mediated selective conversion of CO_2_ to CO at low overpotentials. Science.

[3] Qiao JL, Liu YY, Hong F, Zhang JJ (2014). A review of catalysts for the electroreduction of carbon dioxide to produce low-carbon fuels. Chem. Soc. Rev..

[4] Gao S (2016). Partially oxidized atomic cobalt layers for carbon dioxide electroreduction to liquid fuel. Nature.

[5] Liu M (2016). Enhanced electrocatalytic CO_2_ reduction via field-induced reagent concentration. Nature.

[6] Rogers C (2017). Synergistic enhancement of electrocatalytic CO_2_ reduction with gold nanoparticles embedded in functional graphene nanoribbon composite electrodes. J. Am. Chem. Soc..

[7] Lu Q (2014). A selective and efficient electrocatalyst for carbon dioxide reduction. Nat. Commun..

[8] Kim C (2015). Achieving selective and efficient electrocatalytic activity for CO_2_ reduction using immobilized silver nanoparticles. J. Am. Chem. Soc..

[9] Ma M, Trzesniewski BJ, Xie J, Smith WA (2016). Selective and efficient reduction of carbon dioxide to carbon monoxide on oxide-derived nanostructured silver electrocatalysts. Angew. Chem., Int. Ed..

[10] Gao DF (2015). Size-Dependent electrocatalytic reduction of CO_2_ over Pd nanoparticles. J. Am. Chem. Soc..

[11] Zhu W (2014). Active and selective conversion of CO_2_ to CO on ultrathin Au nanowires. J. Am. Chem. Soc..

[12] Varela AS (2015). Metal-doped nitrogenated carbon as an efficient catalyst for direct CO_2_ electroreduction to CO and hydrocarbons. Angew. Chem. Int. Ed..

[13] Ju W (2017). Understanding activity and selectivity of metal-nitrogen-doped carbon catalysts for electrochemical reduction of CO_2_. Nat. Commun..

[14] Pan FP, Deng W, Justiniano C, Li Y (2018). Identification of champion transition metals centers in metal and nitrogencodoped carbon catalysts for CO_2_ reduction. Appl. Catal. B: Environ..

[15] Moller, T. et al. Efficient CO_2_ to CO electrolysis on solid Ni-N-C catalysts at industrial current densities. *Energy Environ. Sci*. **12**, 640–647 (2019).

[16] Han YH (2018). Electronic structure engineering to boost oxygen reduction activity by controlling the coordination of the central metal. Energy Environ. Sci..

[17] Kou ZK (2017). Molybdenum carbide-derived chlorine-doped ordered mesoporous carbon with few-layered graphene walls for energy storage applications. ACS Appl. Mater. Interfaces.

[18] Kabir S, Artyushkova K, Serov A, Kiefer B, Atanassov P (2016). Binding energy shifs for nitrogen-containing graphene-based electrocatalysts-experiments and DFT calculations. Surf. Interface Anal..

[19] Lin Y-C (2015). Structural and chemical dynamics of pyridinic-nitrogen defects in graphene. Nano Lett..

[20] Yang HB (2018). Atomically dispersed Ni(I) as the active site for electrochemical CO_2_ reduction. Nat. Energy.

[21] Avakyan LA (2013). Atomic structure of nickel phthalocyanine probed by X-ray absorption spectroscopy and density functional simulations. Opt. Spectrosc..

[22] Li JZ (2018). Atomically dispersed manganese catalysts for oxygen reduction in proton-exchange membrane fuel cells. Nat. Catal..

[23] Yang Y (2018). O-, N-Atoms-coordinated Mn cofactors within a graphene framework as bioinspired oxygen reduction reaction electrocatalysts. Adv. Mater..

[24] Zhao SL (2016). Ultrathin metal-organic framework nanosheets for electrocatalytic oxygen evolution. Nat. Energy.

[25] Domenick FL, Timothy A (2014). Mn K-edge X-ray absorption studies of oxo- and hydroxo-manganese(IV) complexes: experimental and theoretical insights into pre-edge properties. Inorg. Chem..

[26] Fei HL (2018). General synthesis and definitive structural identification of MN_4_C_4_ single-atom catalysts with tunable electrocatalytic activities. Nat. Catal..

[27] Li QH (2018). Fe Isolated single atoms on S, N codoped carbon by copolymer pyrolysis strategy for highly efficient oxygen reduction reaction. Adv. Mater..

[28] Liu W (2017). Single-site active cobalt-based photocatalyst with long carriers lifetime for spontaneous overall water splitting. Angew. Chem. Int. Ed..

[29] Li XG (2017). Exclusive Ni-N4 sites realize near-unity CO selectivity for electrochemical CO_2_ reduction. J. Am. Chem. Soc..

[30] Marken, F., Neudeck, A. & Bond, A. M. *Electroanalytical Methods: Guide to Experiments and Applications* (ed. Scholz, F.) 57–106 (Springer, Berlin, 2010).

[31] Yang F (2018). Highly efficient CO_2_ electroreduction on ZnN_4_-based single-atom catalyst. Angew. Chem. Int. Ed..

[32] Lin S (2015). Covalent organic frameworks comprising cobalt porphyrins for catalytic CO_2_ reduction in water. Science.

[33] Zhang X (2017). Highly selective and active CO_2_ reduction electro-catalysts based on cobalt phthalocyanine/carbon nanotube hybrid structures. Nat. Commun..

[34] Han N (2017). Supported cobalt polyphthalocyanine for high-performance electrocatalytic CO_2_ reduction. Chem.

[35] Wang XQ (2018). Regulation of coordination number over single Co sites: triggering the efficient electroreduction of CO_2_. Angew. Chem. Int. Ed..

[36] Pan Y (2018). Design of single-atom Co-N_5_ catalytic site: a robust electrocatalyst for CO_2_ reduction with nearly 100% CO selectivity and remarkable stability. J. Am. Chem. Soc..

[37] Kramer WW, McCrory CCL (2016). Polymer coordination promotes selective CO_2_ reduction by cobalt phthalocyanine. Chem. Sci..

[38] Zhao CM (2017). Ionic exchange of metal-organic frameworks to access single nickel sites for efficient electroreduction of CO_2_. J. Am. Chem. Soc..

[39] Zhang CH (2018). Electrochemical CO_2_ reduction with atomic iron-dispersed on nitrogen-doped graphene. Adv. Energy Mater..

[40] Yang J (2018). In-situ thermal atomization to transfer supported metal nanoparticles to surface enriched Ni single atom catalyst. Angew. Chem. Int. Ed..

[41] Tornow CE, Thorson MR, Ma S, Gewirth AA, Kenis PJA (2012). Nitrogen-based catalysts for the electrochemical reduction of CO_2_ to CO. J. Am. Chem. Soc..

[42] Xie JF (2018). Metal-free fluorine interlayer doped carbon electrocatalyst for CO_2_ reduction outcompeting hydrogen evolution. Angew. Chem. Int. Ed..

[43] Ghausi MA (2018). CO_2_ overall splitting by a bifunctional metal-free electrocatalyst. Angew. Chem. Int. Ed..

[44] Menges FS (2016). Capture of CO_2_ by a cationic nickel(I) complex in the gas phase and characterization of the bound, activated CO_2_ molecule by cryogenic ion vibrational predissociation spectroscopy. Angew. Chem. Int. Ed..

[45] Fujita E, Furenlid LR, Renner MW (1997). Direct XANES evidence for charge transfer in Co-CO_2_ complexes. J. Am. Chem. Soc..

[46] Sakaki S (1990). Can carbon dioxide coordinate to a nickel(I) complex? An ab initio MO/SD-CI study. J. Am. Chem. Soc..

[47] Oiu H-J (2015). Nanoporous graphene with single-atom nickel dopants: an efficient and stable catalyst for electrochemical hydrogen production. Angew. Chem. Int. Ed..

[48] Duan X (2017). Metal-free carbon materials for CO_2_ electrochemical reduction. Adv. Mater..

[49] Jia Q (2015). Experimental observation of redox-induced Fe-N switching behavior as a determinant role for oxygen reduction activity. ACS Nano.

[50] Zhou YS (2018). Dopant-induced electron localization drives CO_2_ reduction to C_2_ hydrocarbons. Nat. Chem..

