# Supplementary material for: Manganese acting as a high-performance heterogeneous electrocatalyst in carbon dioxide reduction
Source: Nat Commun. 2019 Jul 5;10:2980. doi: 10.1038/s41467-019-10854-1 (PMC6611886; doi:10.1038/s41467-019-10854-1)
Supplement: Supplementary file 1 — Supplementary Information [file 41467_2019_10854_MOESM1_ESM.pdf]

# Manganese acting as a high-performance heterogeneous electrocatalyst in carbon dioxide reduction

Zhang et al.

# Supplementary Figures

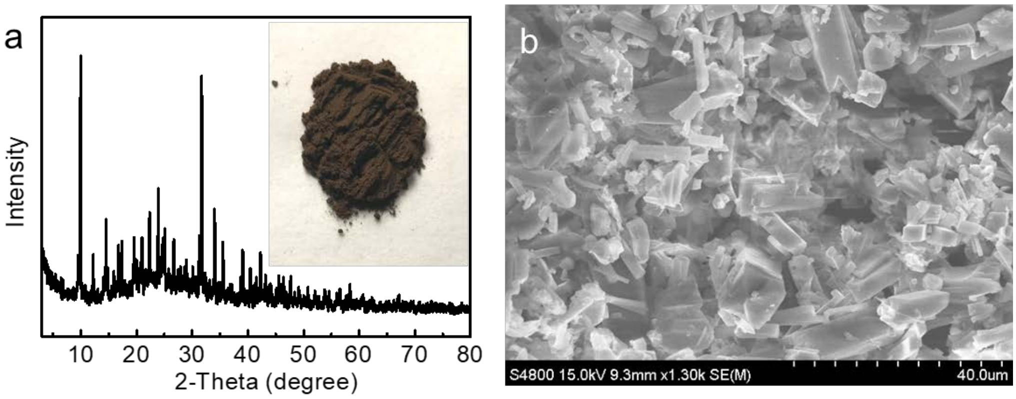


**Supplementary Figure 1 | Characterizations of the Mn-EDA-Cl precursor.** (a) XRD pattern, (b) SEM image. The inset in (a) shows the photograph of Mn-EDA-Cl solid.

a

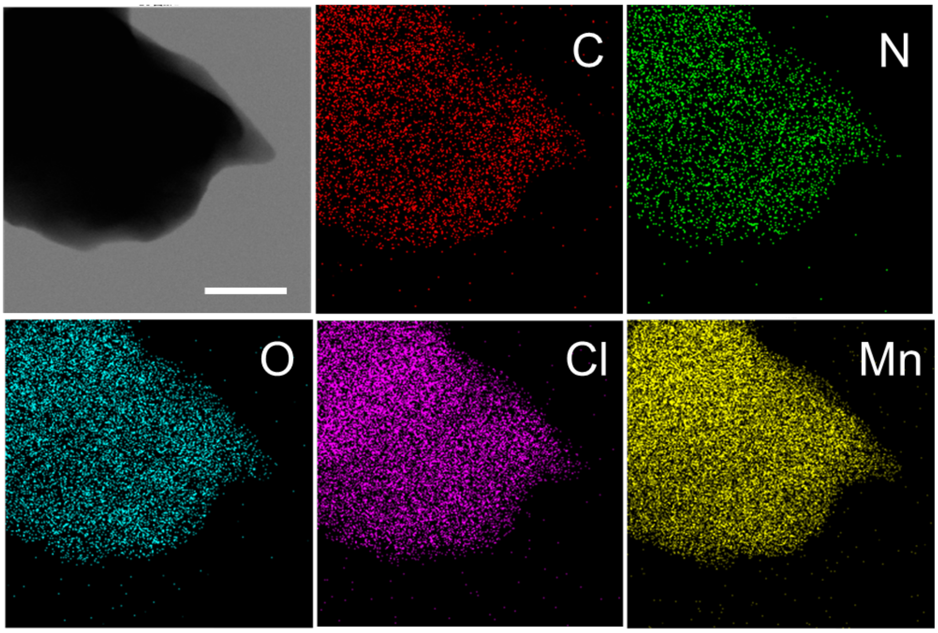


b

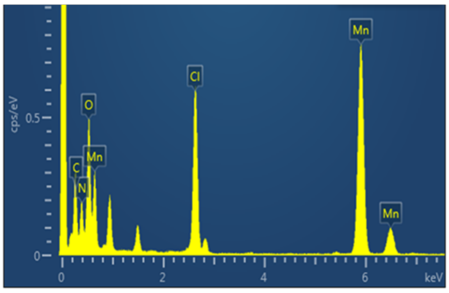


c

| element | wt% | At% |
|---|---|---|
| C | 10.91 | 22.48 |
| N | 10.28 | 18.15 |
| O | 17.11 | 26.45 |
| Cl | 20.75 | 14.48 |
| Mn | 40.95 | 18.44 |
| Total | 100.00 | 100.00 |

**Supplementary Figure 2 | Element composition of Mn-EDA-Cl.** EDS mappings (a), EDS spectrum (b) and corresponding element content of Mn-EDA-Cl (c).

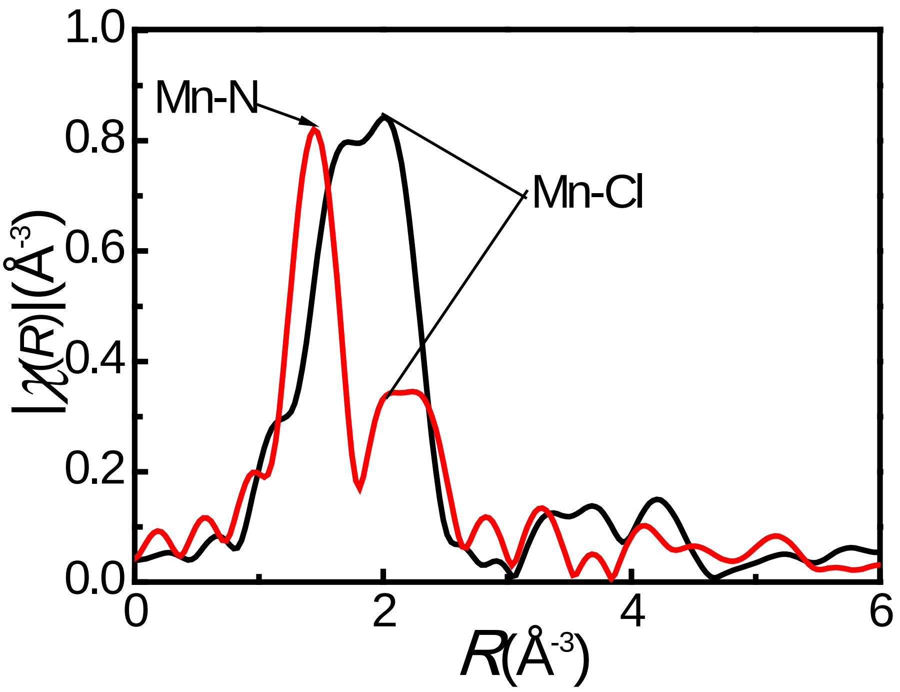


**Supplementary Figure 3 | Fine structure of Mn-EDA-Cl.** Extended X-ray absorption fine structure (EXAFS) spectra of $MnCl_2$ (black) and Mn-EDA-Cl (red). The two peaks around 1.45 Å and 2.0 Å in the first shell of Mn-EDA-Cl correspond to Mn-N and Mn-Cl scatterings, respectively. It suggests that Mn coordinated with N of EDA and Cl in Mn-EDA-Cl.

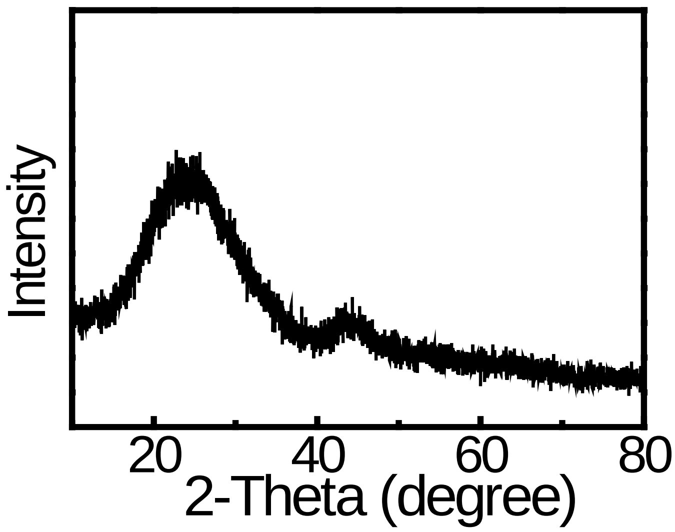


**Supplementary Figure 4 | XRD pattern of (Cl, N)-Mn/G.** The diffractions at 24.0° and 44.1° correspond to (002) and (004) of graphitic carbon layer, respectively.

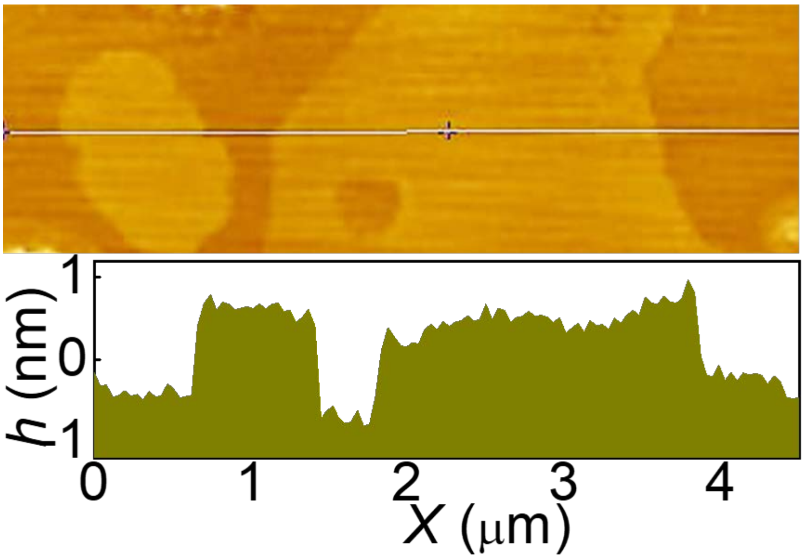


**Supplementary Figure 5 | AFM image of (Cl, N)-Mn/G**.

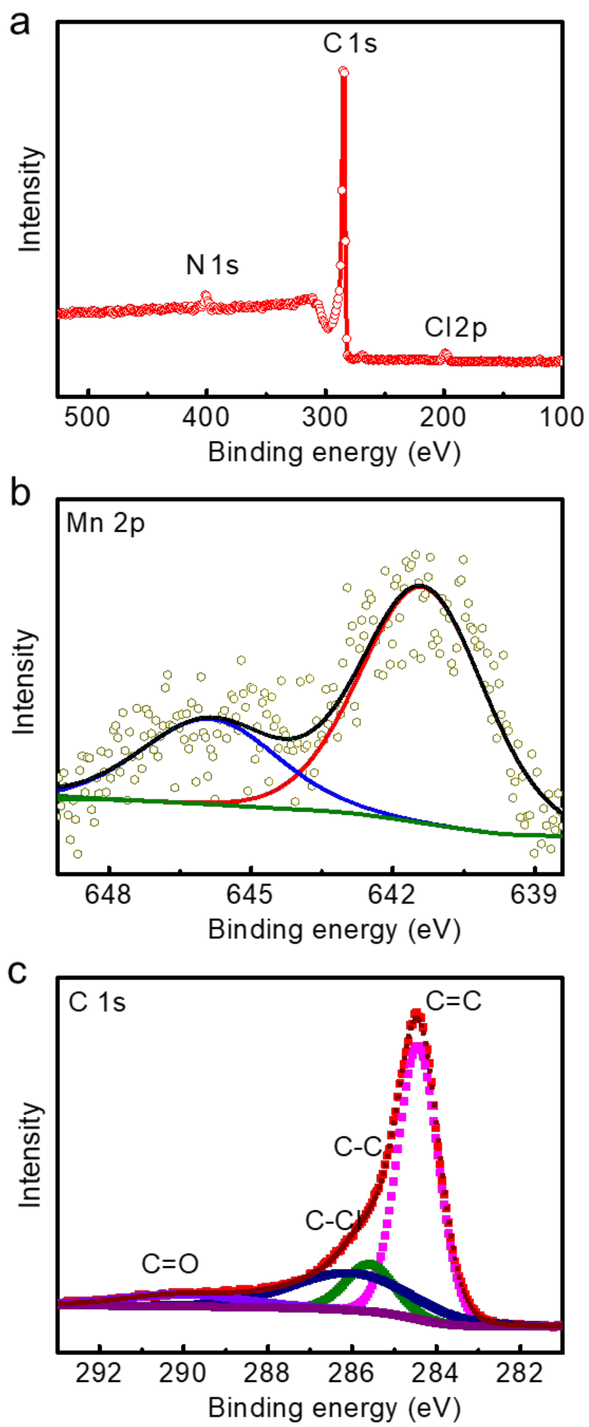


**Supplementary Figure 6 | XPS characterizations for (Cl, N)-Mn/G.** Full XPS spectrum (a), Mn 2p (b) and C 1s (c) XPS spectra of (Cl, N)-Mn/G. The Mn 2p XPS shows no metallic Mn species (at ~639.0 eV) in the catalyst, which is in accord with the atomically dispersed Mn. The peak at 286.2 eV in C 1s XPS spectrum corresponds to C-Cl specie, which is consistent with the C-Cl species reflected by Cl 2p XPS spectrum in Fig 2b.

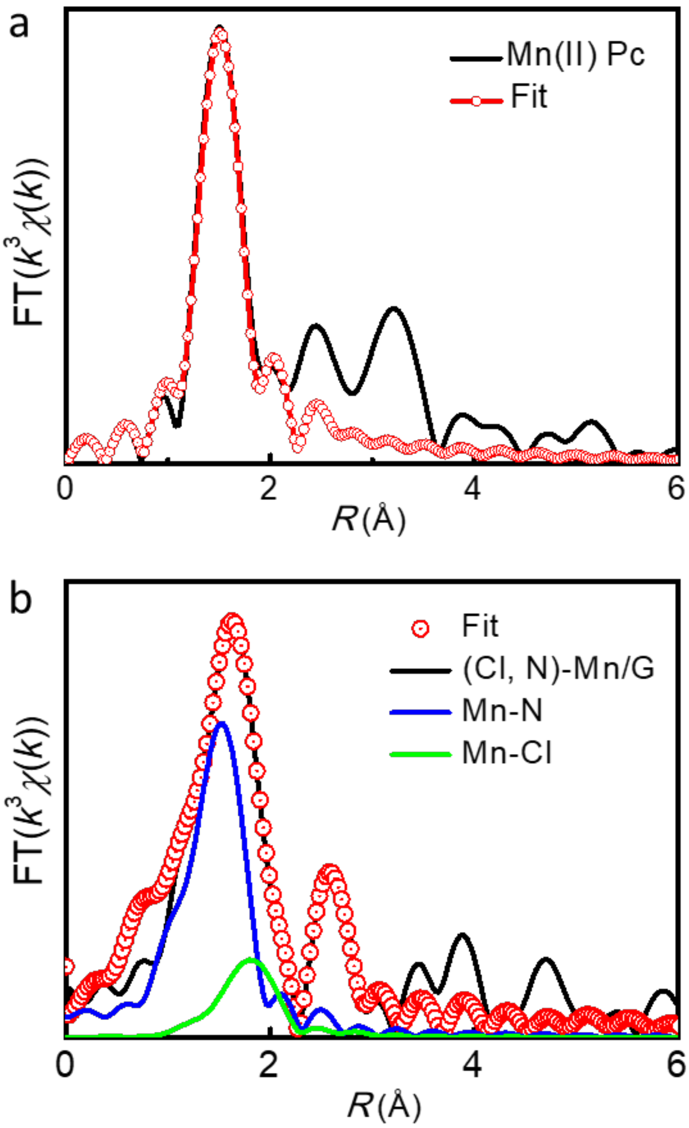


**Supplementary Figure 7 | EXAFS fitting results of Mn(II) Pc and (Cl, N)-Mn/G.** EXAFS fitting curves of Mn(II) Pc (a) and (Cl, N)-Mn/G (b) in *R* space. The coordination environment for (Cl, N)-Mn/G is investigated by quantitative least-squares EXAFS curve-fitting. In the EXAFS fitting, two scattering paths (Mn-N and Mn-Cl) were applied[1-5]. The best EXAFS fitting result clearly shows that the main peak at R space derived from Mn-N and Mn-Cl scatterings. The corresponding structure parameters are listed in the Supplementary Table 2.

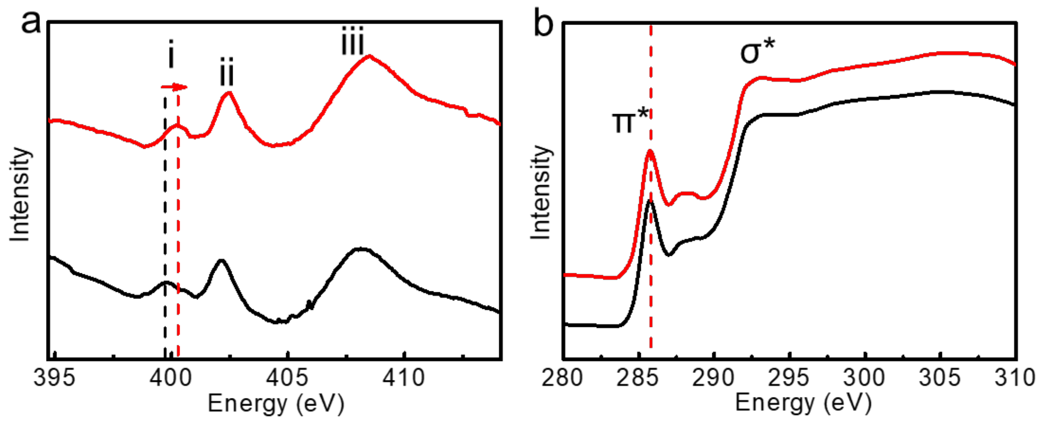


**Supplementary Figure 8 | Soft XAS of (Cl, N)-Mn/G and N-G.** N K-edge (a) and C K-edge (b) XAS of (Cl, N)-Mn/G (red) and N-G (black). The N K-edge XAS spectra in Supplementary Fig 8a present C-N-C portion (peak i), N-3C bridging (peak ii), and the C-N bond (peak iii). Obviously, peak i of (Cl, N)-Mn/G shifts to higher energy compared with that of N doped graphene, indicating N formed a chemical bond with Mn sites[6]. While the C K-edge XAS spectra in Supplementary Fig 8b show no difference of position between the two samples, excluding formation of the Mn-C bond. Thus, we could conclude single Mn sites bond to N in (Cl, N)-Mn/G.

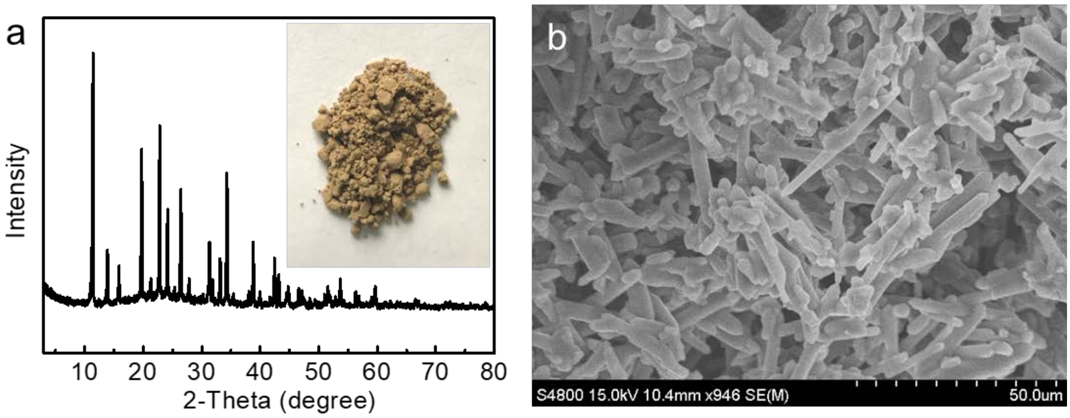


**Supplementary Figure 9 | Characterizations for the Mn-EDA-$NO_3$ precursor.** XRD pattern (a) and SEM image (b) of Mn-EDA-$NO_3$ precursor. The inset in (a) shows the photograph of Mn-EDA-$NO_3$.

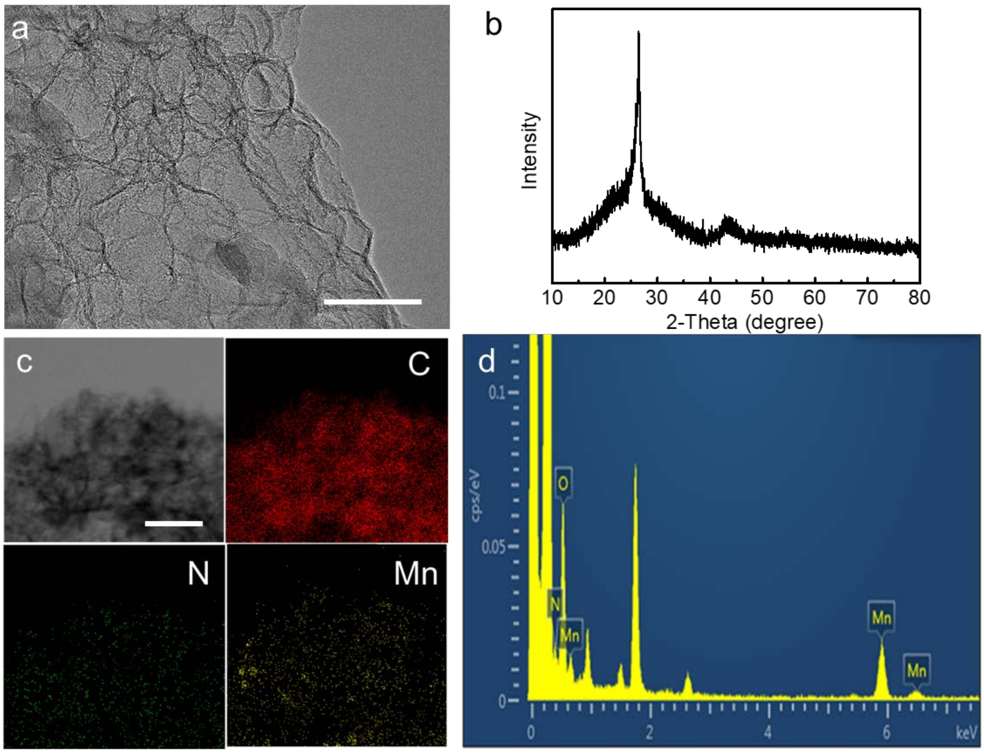


**Supplementary Figure 10 | Characterizations for N-Mn/G.** TEM image (a), XRD pattern (b) and EDS images (c, d) of N-Mn/G. Scare bars, 50 nm in (a) and 200 nm in (c).

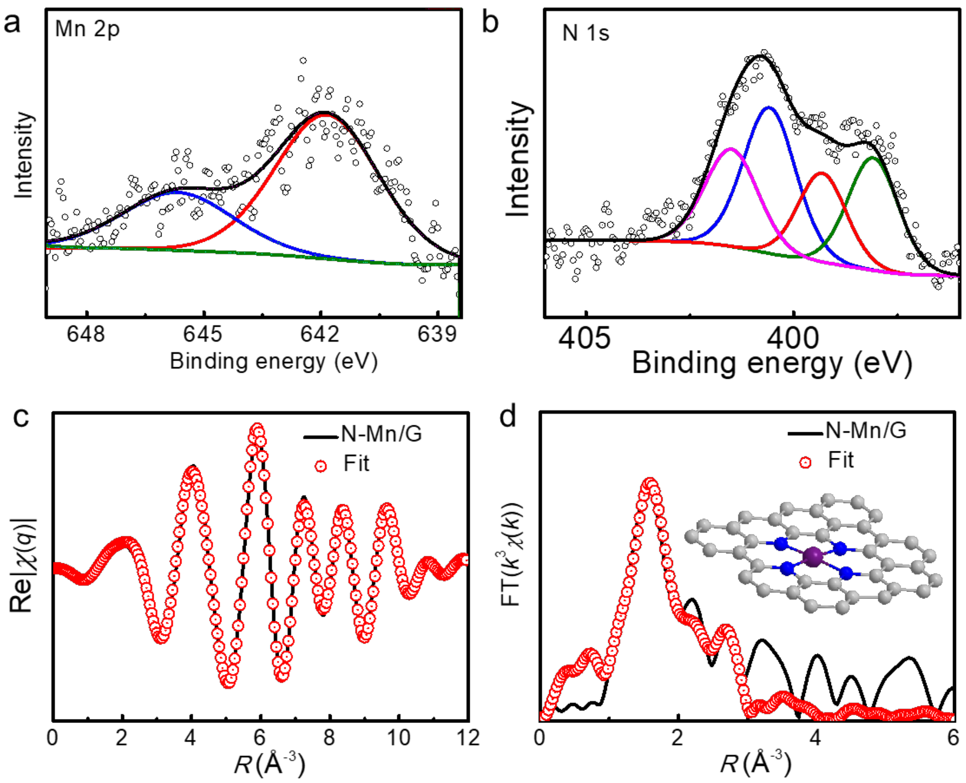


**Supplementary Figure 11 | Fine structures for N-Mn/G.** (a) High-resolution Mn 2p XPS spectra of N-Mn/G. (b) N 1s XPS spectra of N-Mn/G. The N 1s XPS spectrum reveals four N species, including Mn-N species at 399.3 eV, pyridinic N (398.1 eV), pyrrolic N (400.6 eV) and graphitic N (401.5 eV). (c) EXAFS fitting curves of N-Mn/G in *q* space. (d) EXAFS fitting curves of the N-Mn/G in *R* space. Inset: Schematic model of N-Mn/G: Mn (purple), N (blue), and C (gray).

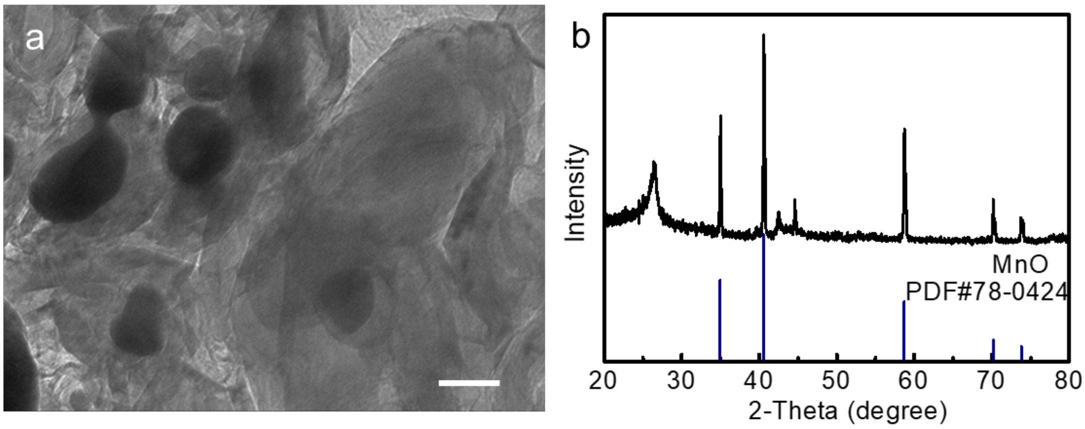


**Supplementary Figure 12 | Characterizations of MnO/G.** TEM image (a) and XRD pattern (b) of MnO/G. Scale bar, 50 nm.

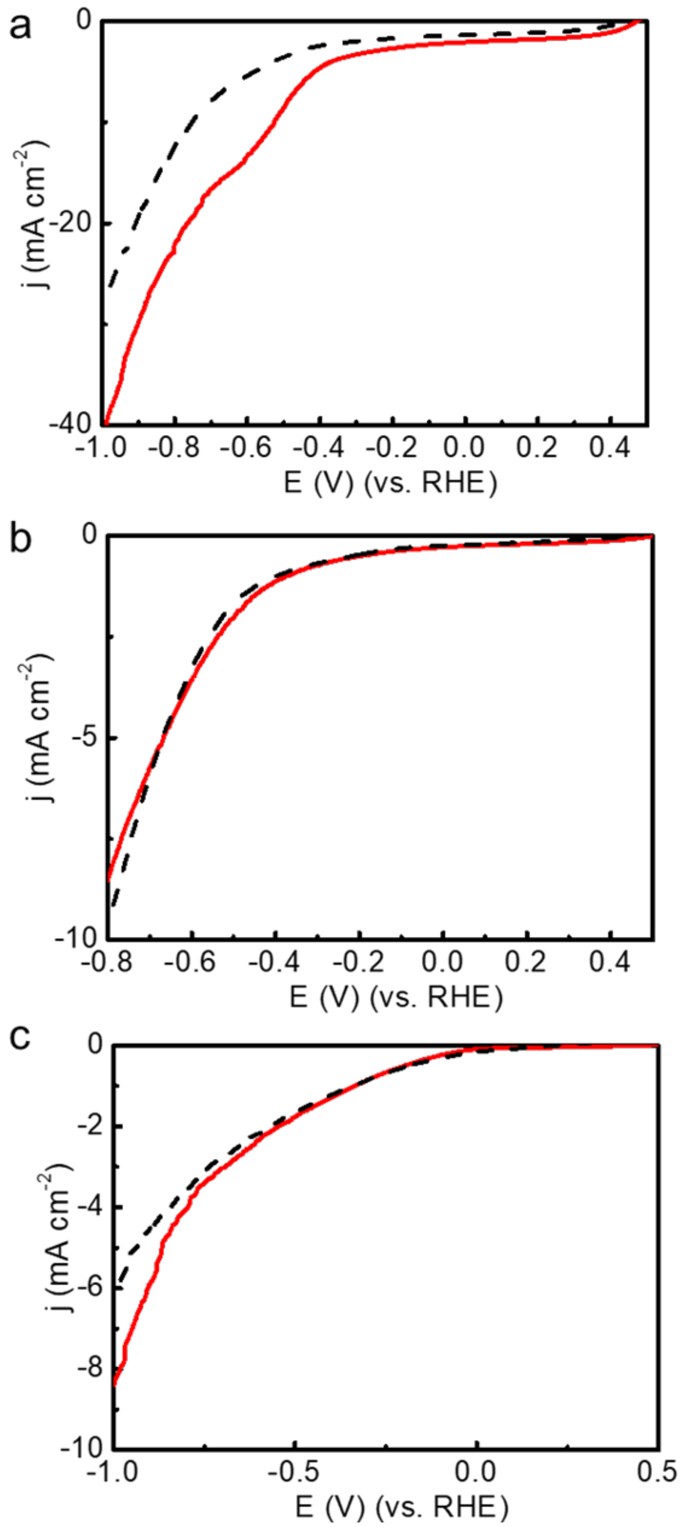


**Supplementary Figure 13 | Electrochemical $CO_2$RR performance on (Cl, N)-Mn/G and control samples.** LSV curves of (Cl, N)-Mn/G (a), N-Mn/G (b) and MnO/G (c) electrodes in Ar (black) and $CO_2$ (red) saturated 0.5 M $KHCO_3$ at a scan rate of 10 mV $s^{-1}$.

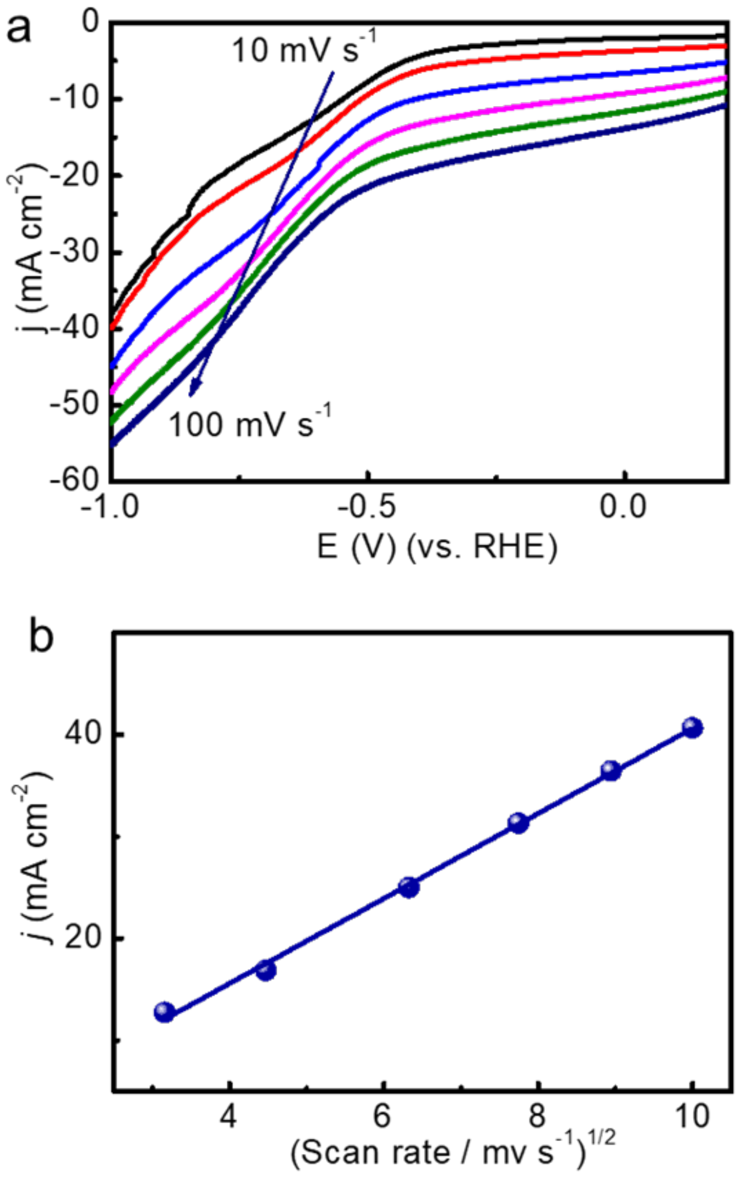


**Supplementary Figure 14 | Electrochemical $CO_2$RR performance on (Cl, N)-Mn/G.** Scan rate dependent LSV response (a) and peak current of (Cl, N)-Mn/G vs. scan rate$^{1/2}$ (b).

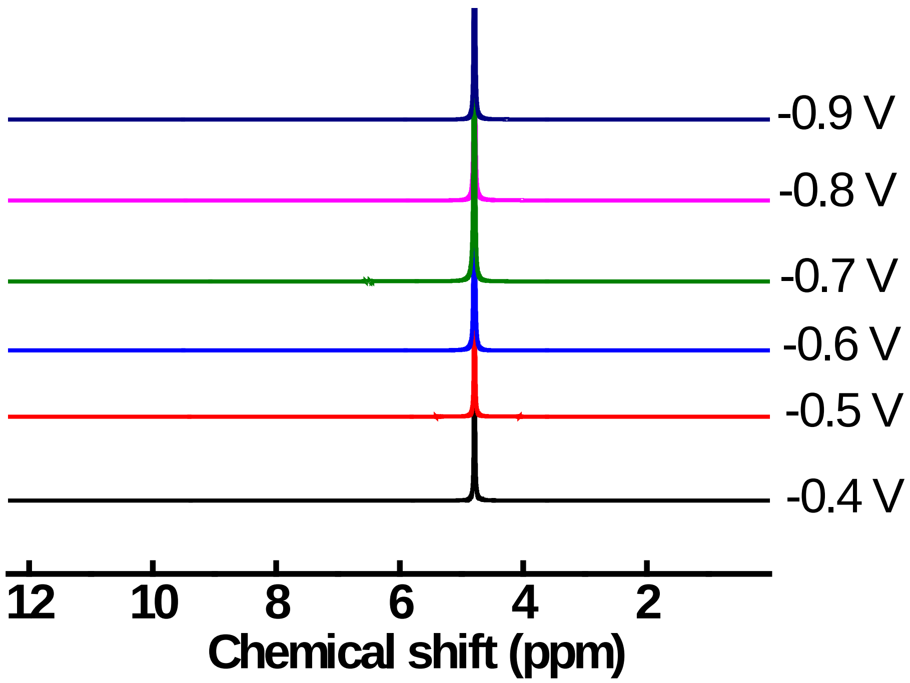


**Supplementary Figure 15 | Liquid products analysis.** $^{1}$H-NMR spectra of the electrolyte after electrolysis using (Cl, N)-Mn/G at various potentials.

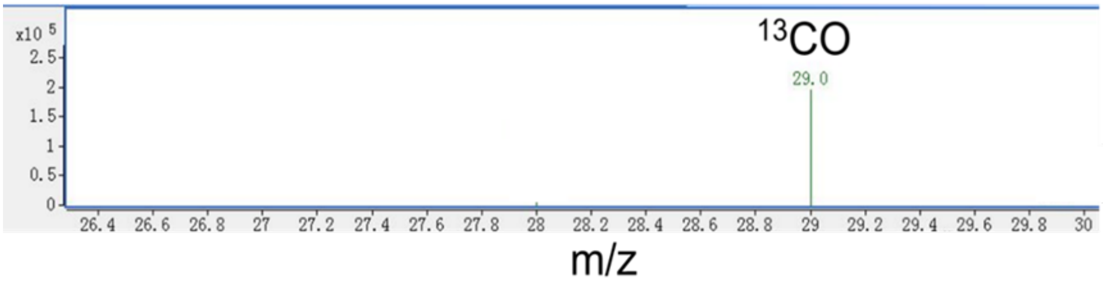


**Supplementary Figure 16 | Mass spectrometry signal of the $CO_2$ reduction products using $^{13}CO_2$ as the feedstock.**

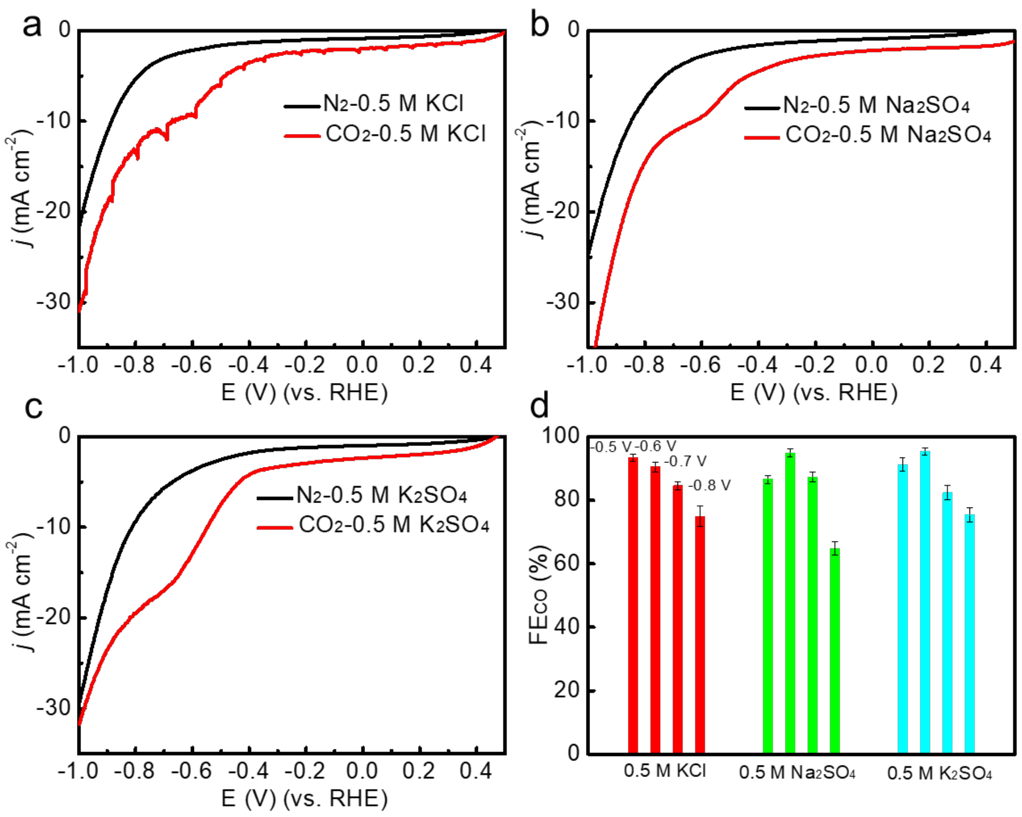


**Supplementary Figure 17 | Electrochemical $CO_2$RR performance on (Cl, N)-Mn/G in different electrolyte.** LSV curves of (Cl, N)-Mn/G in $N_2$ (black) and $CO_2$ (red) saturated 0.5 M KCl (a), $Na_2SO_4$ (b) and $K_2SO_4$ (c) solution and corresponding CO faradaic efficiencies at various applied potentials (d). The pH values of the $N_2$-saturated 0.5 M KCl, $Na_2SO_4$, and $K_2SO_4$ electrolytes were adjusted by acid to be 4.32, 4.18 and 4.23, which are similar to those of the $CO_2$-saturated 0.5 M KCl (4.35), $Na_2SO_4$ (4.15), and $K_2SO_4$ electrolytes (4.26), respectively. As shown in Supplementary Figure 17 a-c, a cathodic peak occurs in the LSV curve of the $CO_2$-saturated electrolyte, which was not observed for the $N_2$-saturated electrolyte. Also, the current density in $CO_2$-saturated electrolyte is much higher than that in $N_2$-saturated electrolyte. The results indicate that the $CO_2$ reduction catalyzed by (Cl, N)-Mn/G indeed contributes to the high current density.

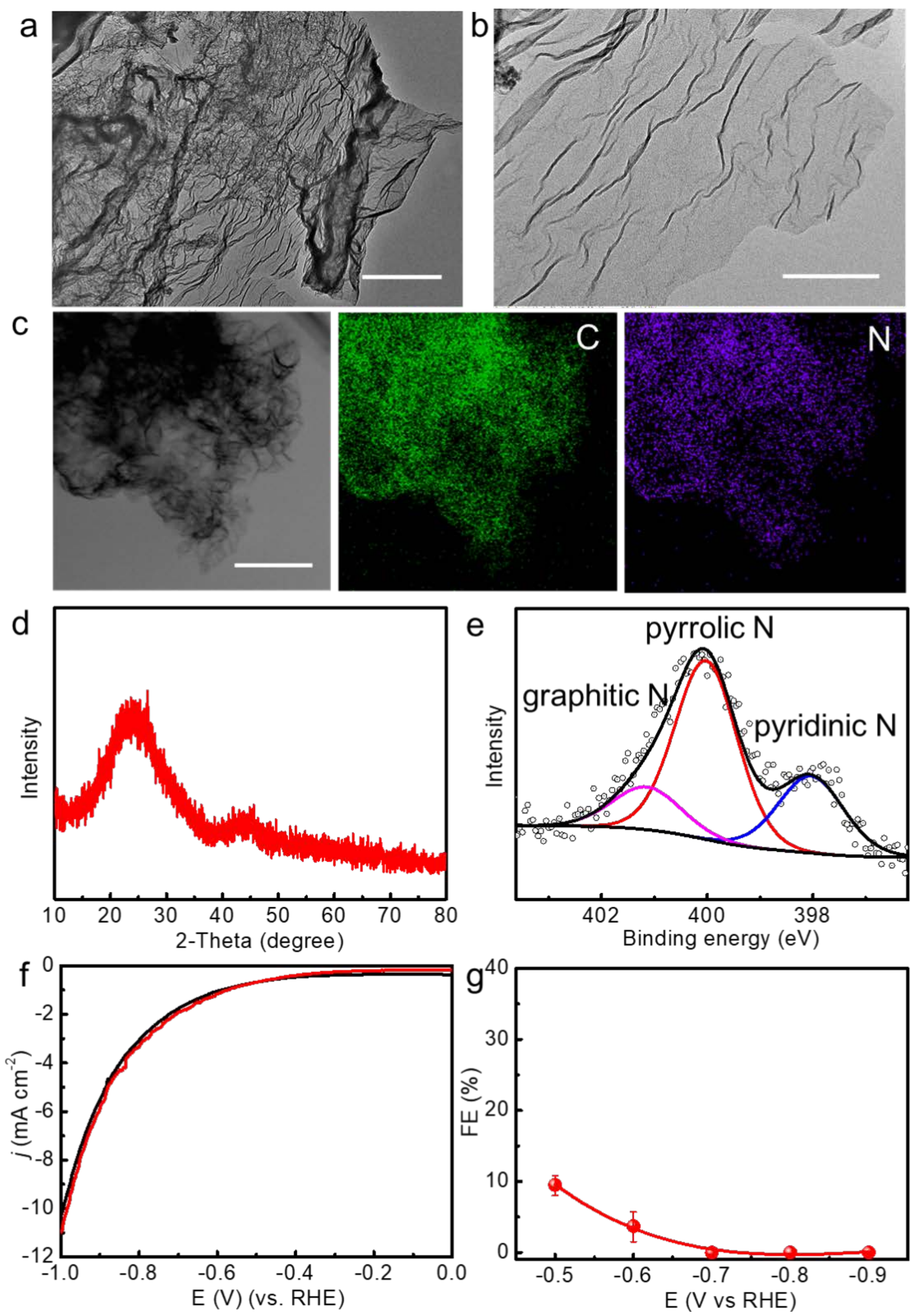


**Supplementary Figure 18 | Characterizations and electrochemical $CO_2$RR performance of N doped graphene.** (a, b) TEM images. (c) EDS image. (d) XRD pattern. (e) N1s XPS spectra. (f) LSV curves in Ar (black) and $CO_2$ (red) saturated 0.5 M $KHCO_3$ at a scan rate of 10 mV $s^{-1}$. (g) CO faradaic efficiencies at various applied potentials. Scale bar, 500 nm in (a), (c) and 200 nm in (b).

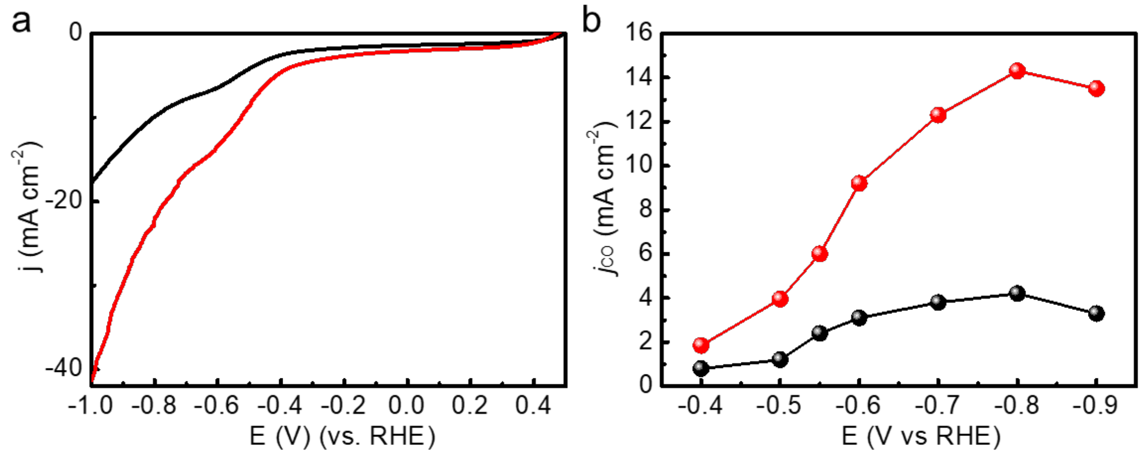


**Supplementary Figure 19 | The $CO_2$RR activity of (Cl, N)-Mn/G after poisoning by $SCN^-$.** LSV curves (a) and potential-dependent CO partial current density (b) of (Cl, N)-Mn/G before (red) and after (black) poisoning by $SCN^-$ in $CO_2$-saturated 0.5 M $KHCO_3$. The $SCN^-$ can poison metal sites according to literatures[6].

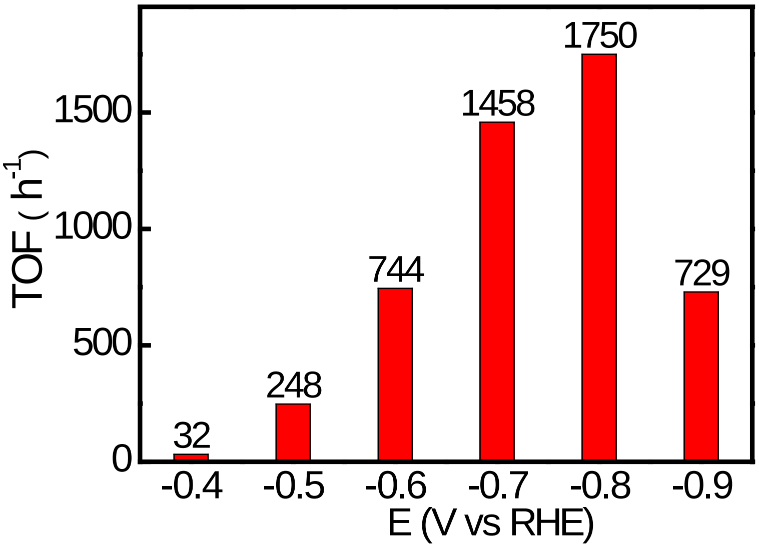


**Supplementary Figure 20 | TOF of N-Mn/G at various potentials.**

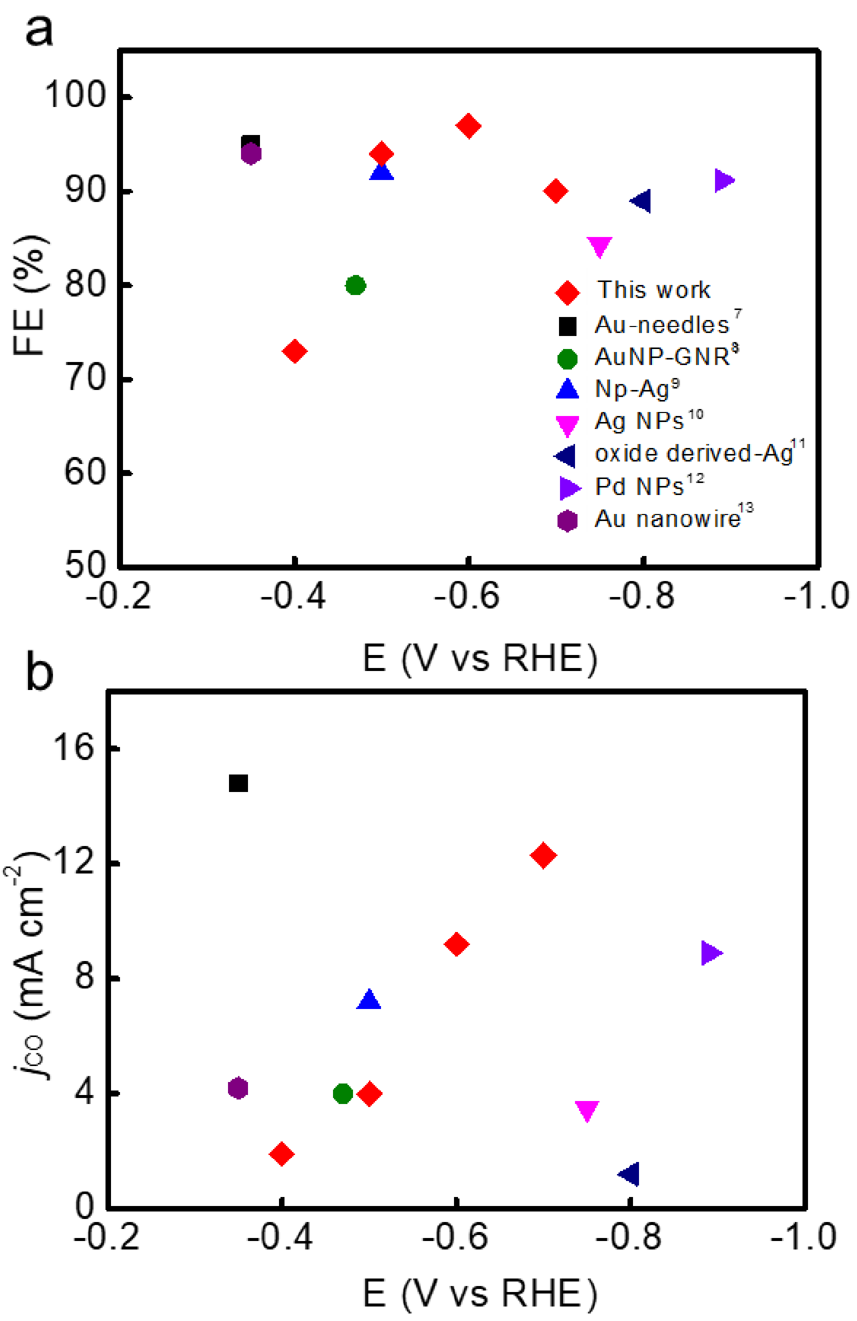


**Supplementary Figure 21 | Comparison of typical noble metal catalysts and (Cl, N)-Mn/G for $CO_2$ electroreduction to CO.** Comparison of typical noble metal catalysts[7-13] and (Cl, N)-Mn/G for $CO_2$ electroreduction to CO in $CO_2$-saturated 0.5 M $KHCO_3$ solution with a catalyst loading of 0.5 mg $cm^{-2}$ for (Cl, N)-Mn/G. (a) CO faradaic efficiency and (b) CO partial current density normalized to geometric area.

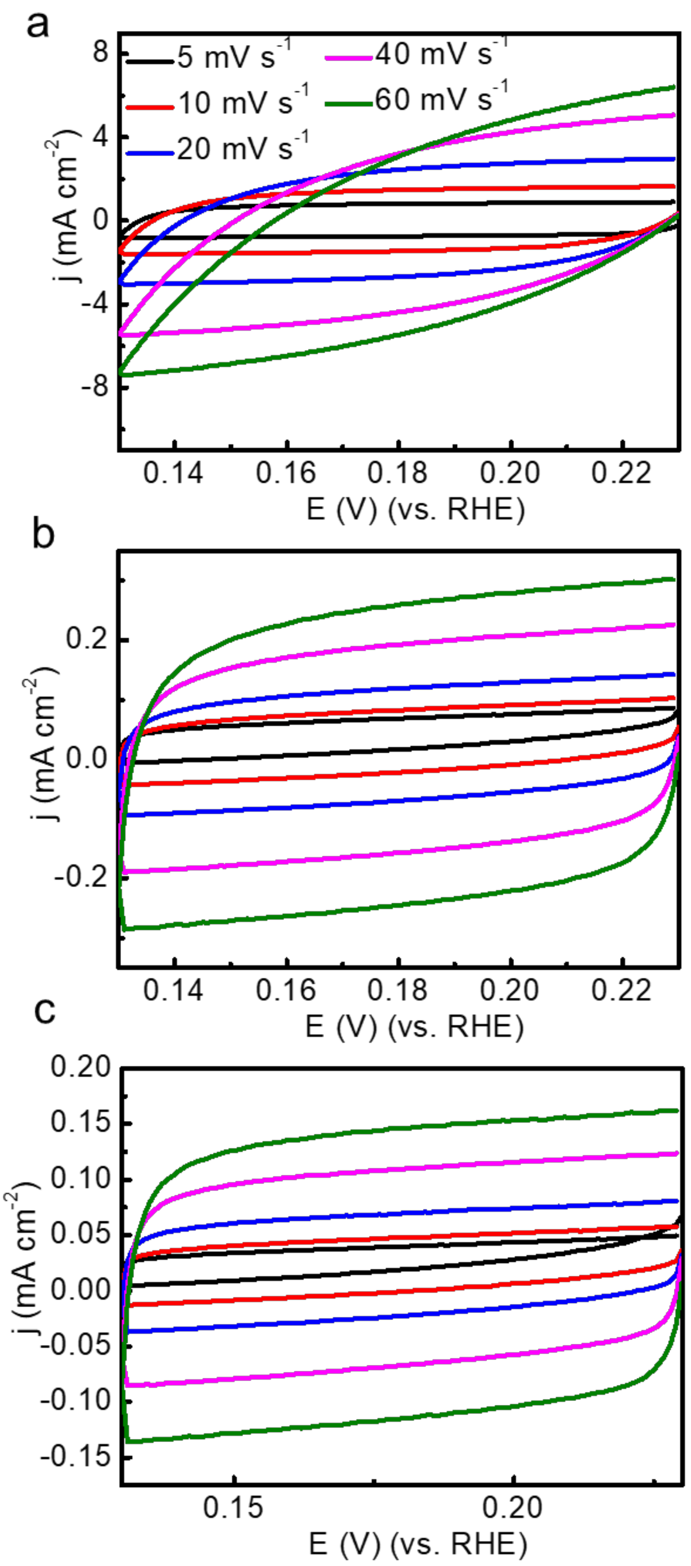


**Supplementary Figure 22 | ECSA test.** Cyclic voltammograms of (Cl, N)-Mn/G (a), N-Mn/G (b) and MnO/G (c) with different scan rate.

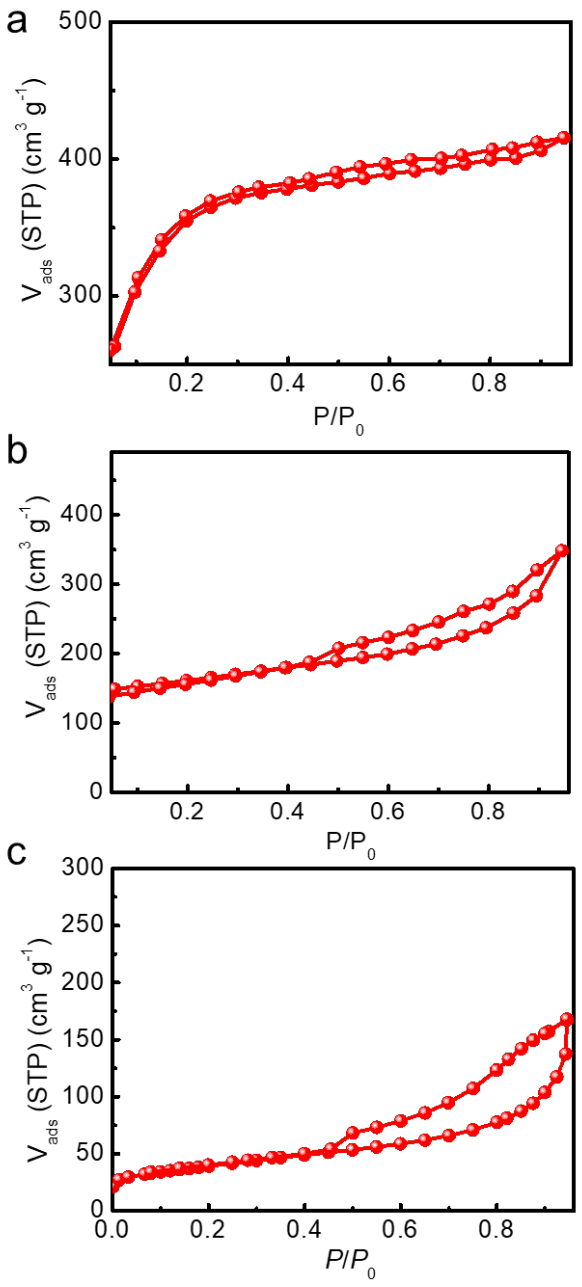


**Supplementary Figure 23 | $N_2$ adsorption-desorption isotherms of (Cl, N)-Mn/G (a), N-Mn/G (b) and MnO/G (c).** The specific surface area of (Cl, N)-Mn/G, N-Mn/G and MnO/G is 1192, 425 and 156 $m^2$ $g^{-1}$, respectively.

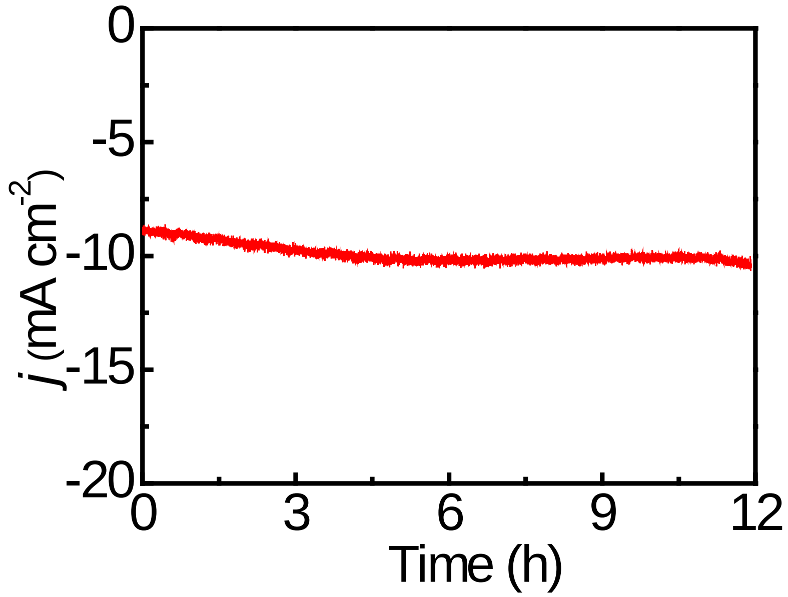


**Supplementary Figure 24 | Stability test.** Amperometric (i ~ t) stability at -0.6 V (*vs* RHE) for 12 h.

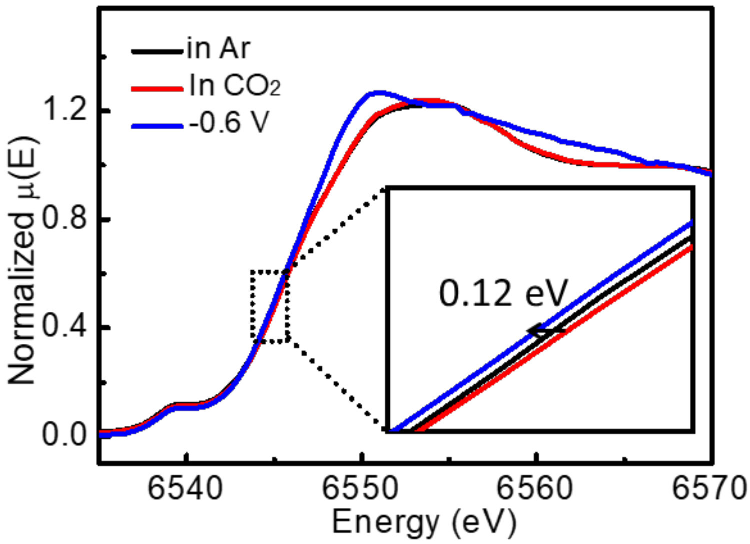


**Supplementary Figure 25 | Normalized XANES of N-Mn/G catalyst under various conditions (inset is the magnified image)**.

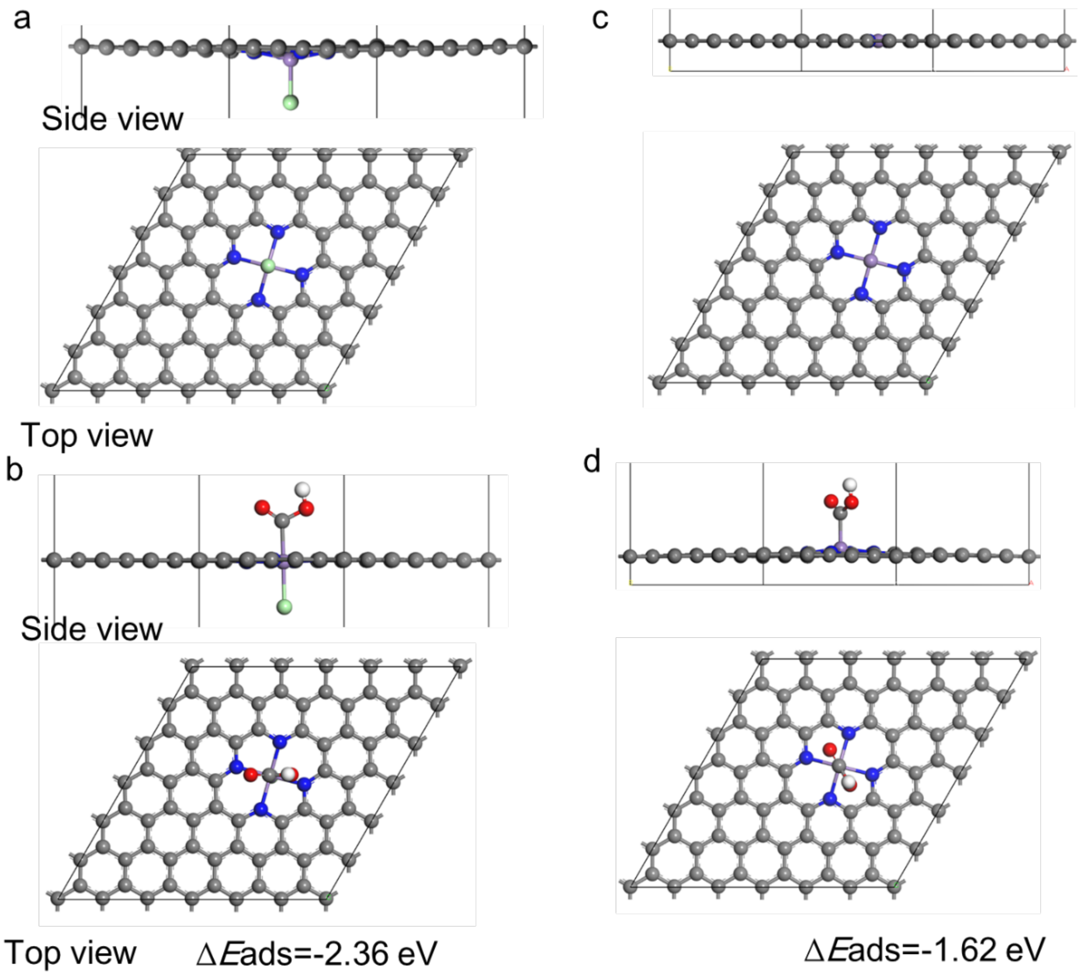


**Supplementary Figure 26 | DFT data of the optimized configurations and binding energy of intermediates on catalysts.** (a) Optimized structure for (Cl, N)-Mn/G. (b) Optimized structure for the intermediate COOH* adsorbed on (Cl, N)-Mn/G. (c) Optimized structure for N-Mn/G. (d) Optimized structure for the intermediate COOH* adsorbed on N-Mn/G.

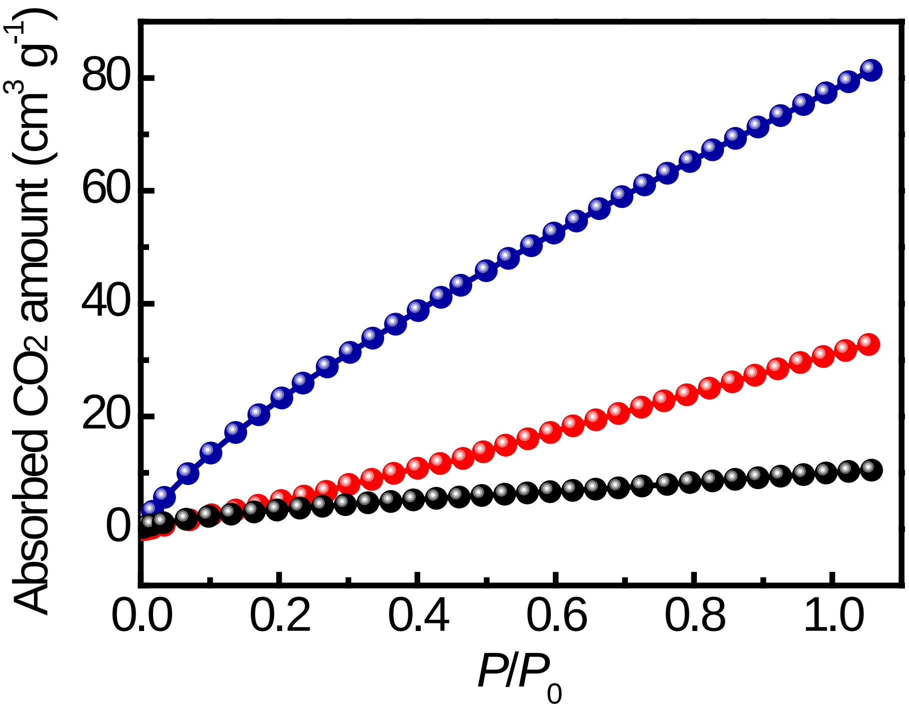


**Supplementary Figure 27 | $CO_2$ adsorption test.** $CO_2$ adsorption isotherm for (Cl, N)-Mn/G (blue), N-Mn/G (red) and MnO/G (black). (Cl, N)-Mn/G exhibits a larger adsorption capacity (77.4 $cm^3$ $g^{-1}$) than that of N-Mn/G (30.6 $cm^3$ $g^{-1}$) and MnO/G (9.9 $cm^3$ $g^{-1}$).

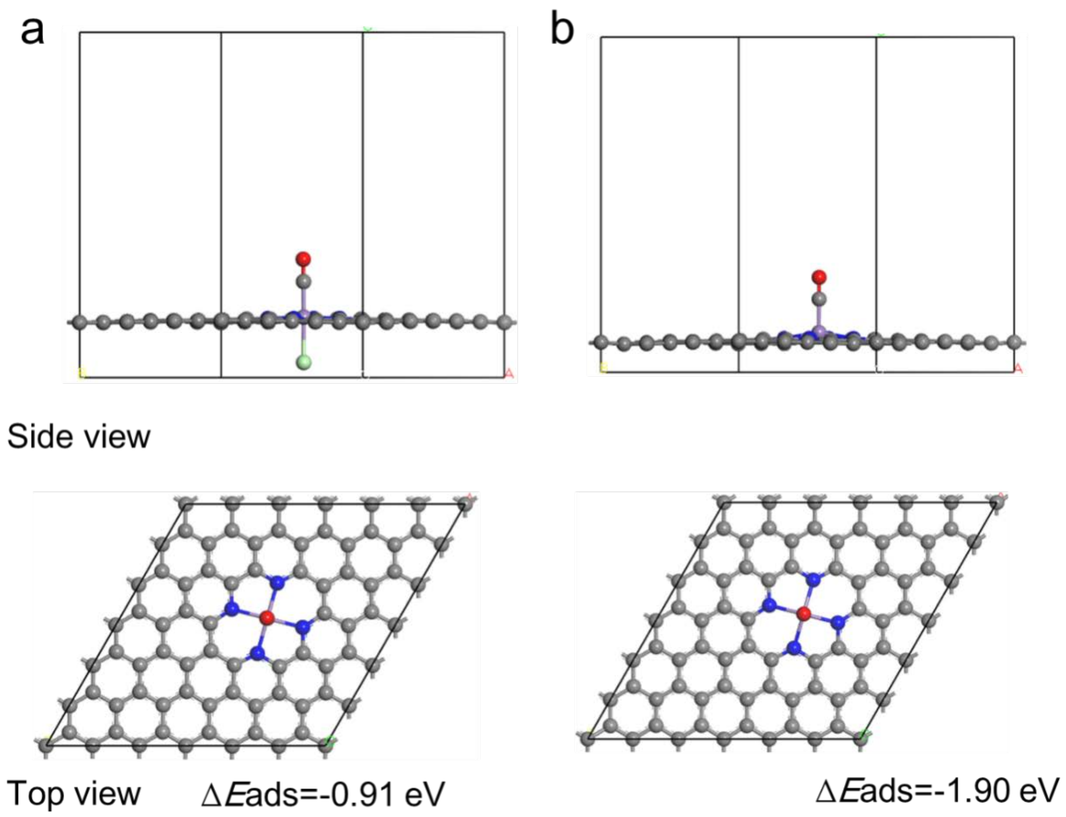


**Supplementary Figure 28 | DFT data of the optimized configurations and binding energy of intermediates on catalysts.** (a) Optimized structure for the intermediate CO* adsorbed on (Cl, N)-Mn/G. (b) Optimized structure for the intermediate CO* adsorbed on N-Mn/G.

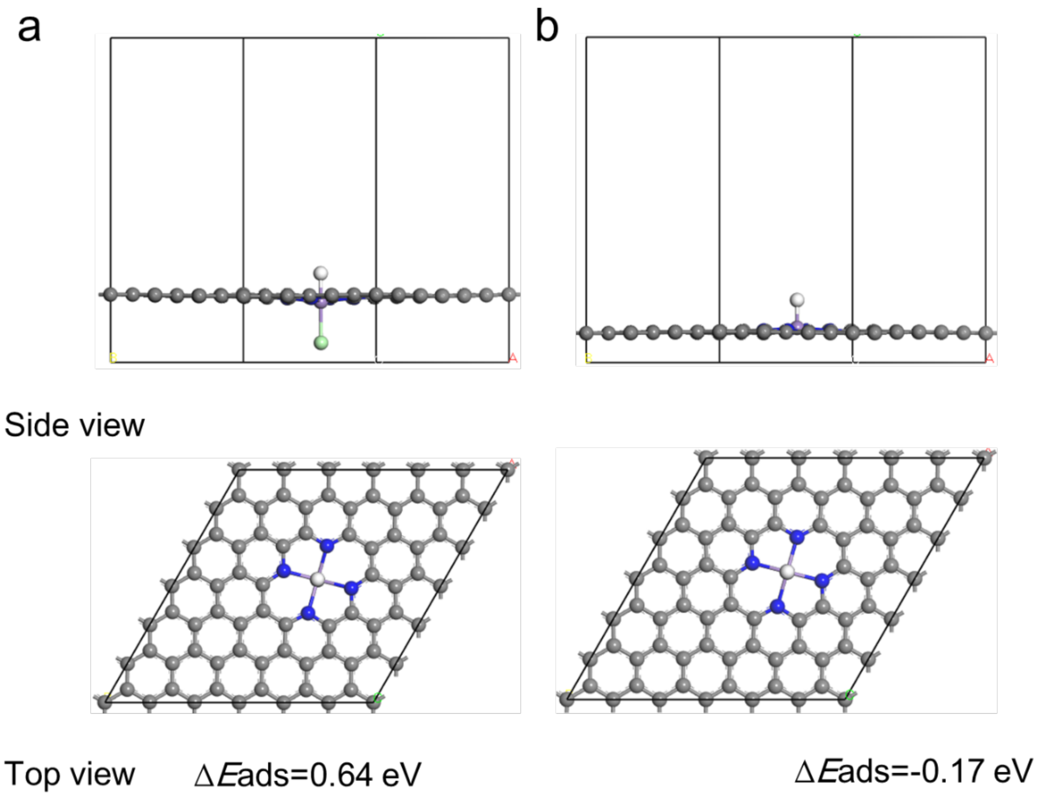


**Supplementary Figure 29 | DFT data of the optimized configurations and binding energy of intermediates on catalysts.** (a) Optimized structure for the intermediate H* adsorbed on (Cl, N)-Mn/G. (b) Optimized structure for the intermediate H* adsorbed on N-Mn/G.

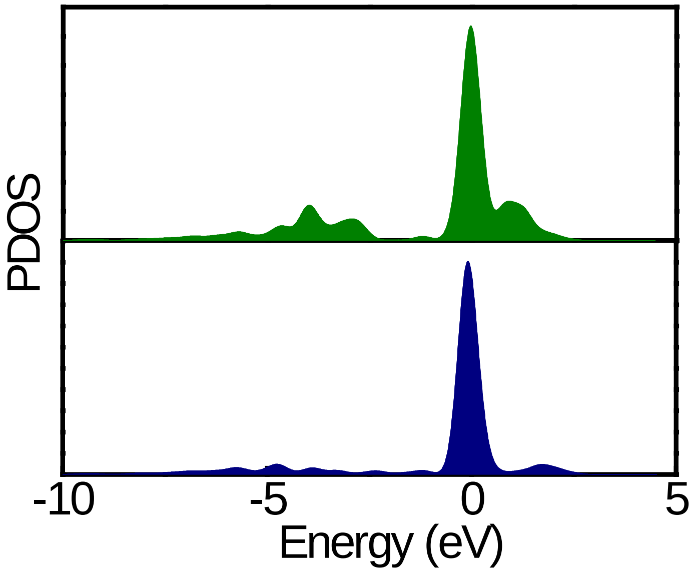


**Supplementary Figure 30 | Electronic structure of original (Cl, N)-Mn/G and N-Mn/G.** d-projected DOS of Mn for original (Cl, N)-Mn/G (top) and N-Mn/G (bottom), respectively.

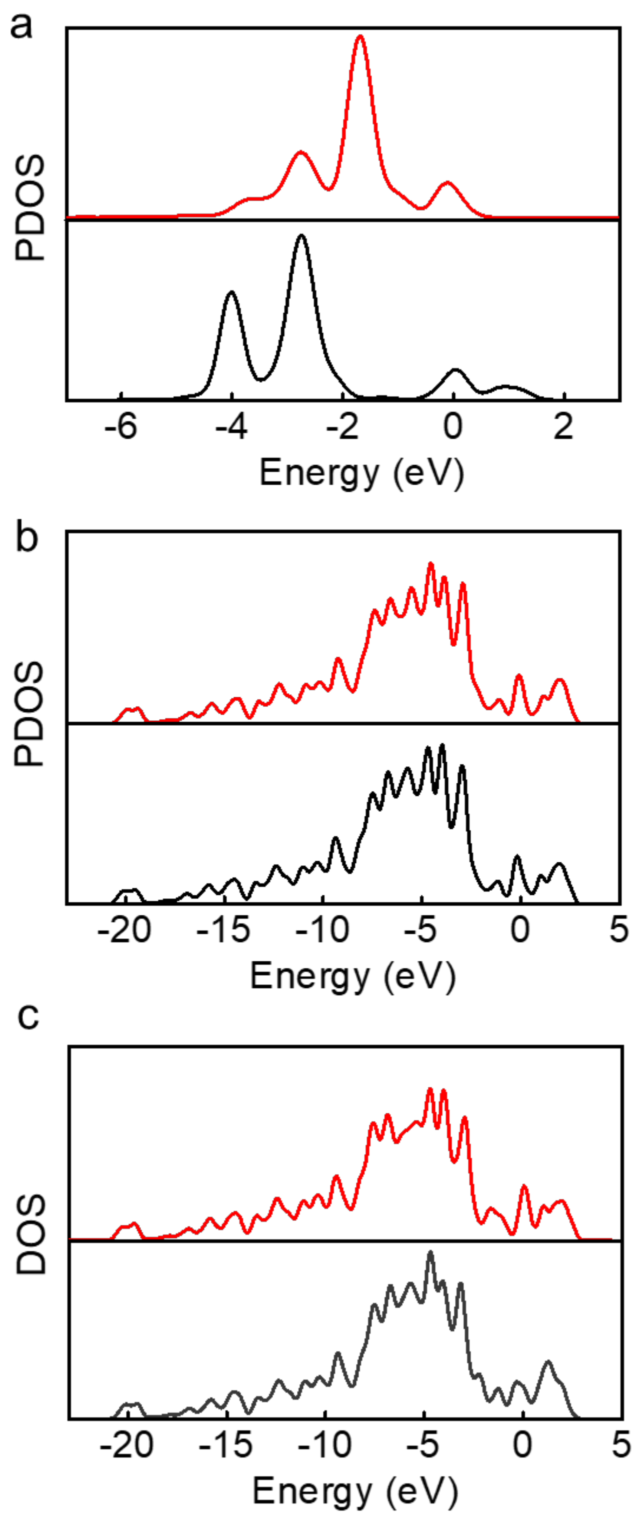


**Supplementary Figure 31 | Electronic structure of Cl and N in corresponding structures.** (a) p-projected DOS of Cl for original (Cl, N)-Mn/G (black) and COOH* adsorbed (Cl, N)-Mn/G (red), respectively. (b) p-projected DOS of N for original (Cl, N)-Mn/G (black) and COOH* adsorbed (Cl, N)-Mn/G (red), respectively. (c) p-projected DOS of N for original N-Mn/G (black) and COOH* adsorbed N-Mn/G

(red), respectively. The d-projected DOS of Mn in (Cl, N)-Mn/G shows a higher DOS around $E_F$ than that in N-Mn/G (Supplementary Fig. 30), corresponding to the contribution of Cl coordination. The p-projected DOS (Supplementary Fig. 31) of Cl in (Cl, N)-Mn/G displays an evident upshift after interacting with COOH*, while the p-projected DOS of N shows no obvious change. These results strongly indicate the important role of coordinated Cl for modulating the electronic state for the single Mn active center to improved reactivity.

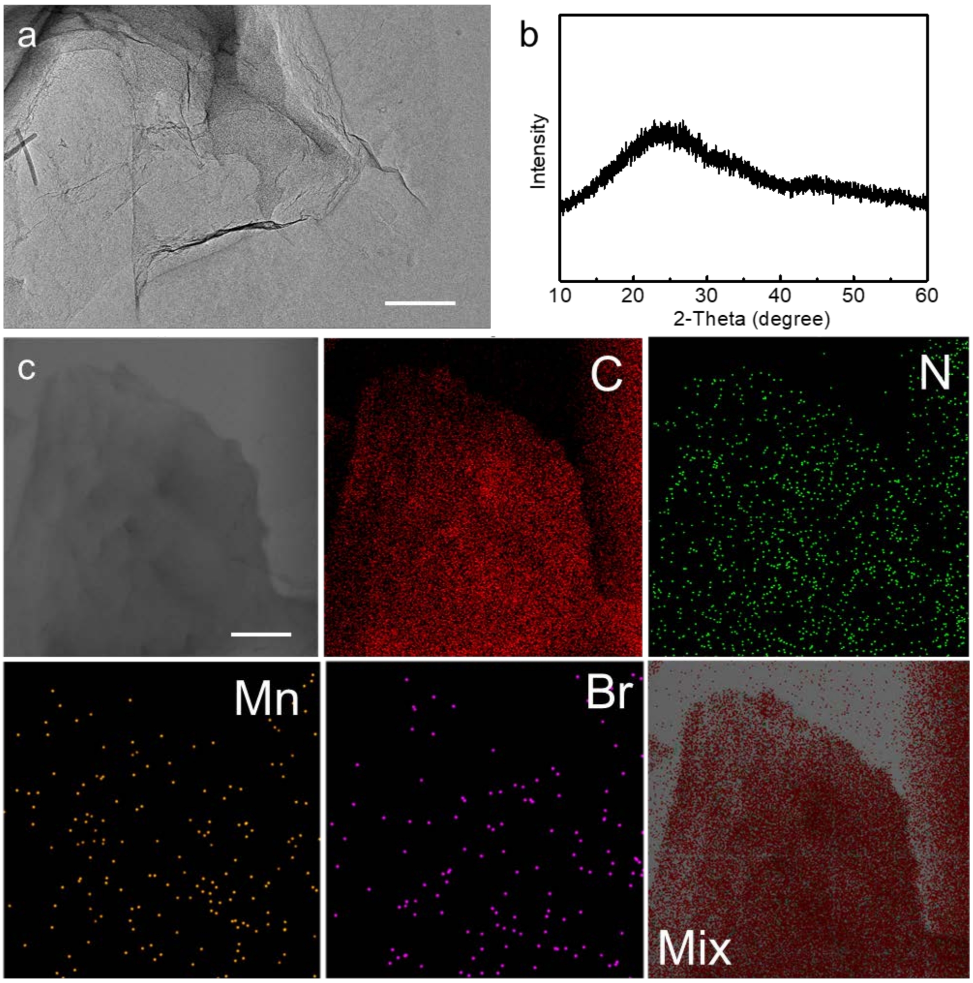


**Supplementary Figure 32 | Structural characterizations of (Br, N)-Mn/G.** TEM image (a), XRD pattern (b) and EDS images (c) of (Br, N)-Mn/G. Scale bar, 200 nm in (a) and 500 nm in (c).

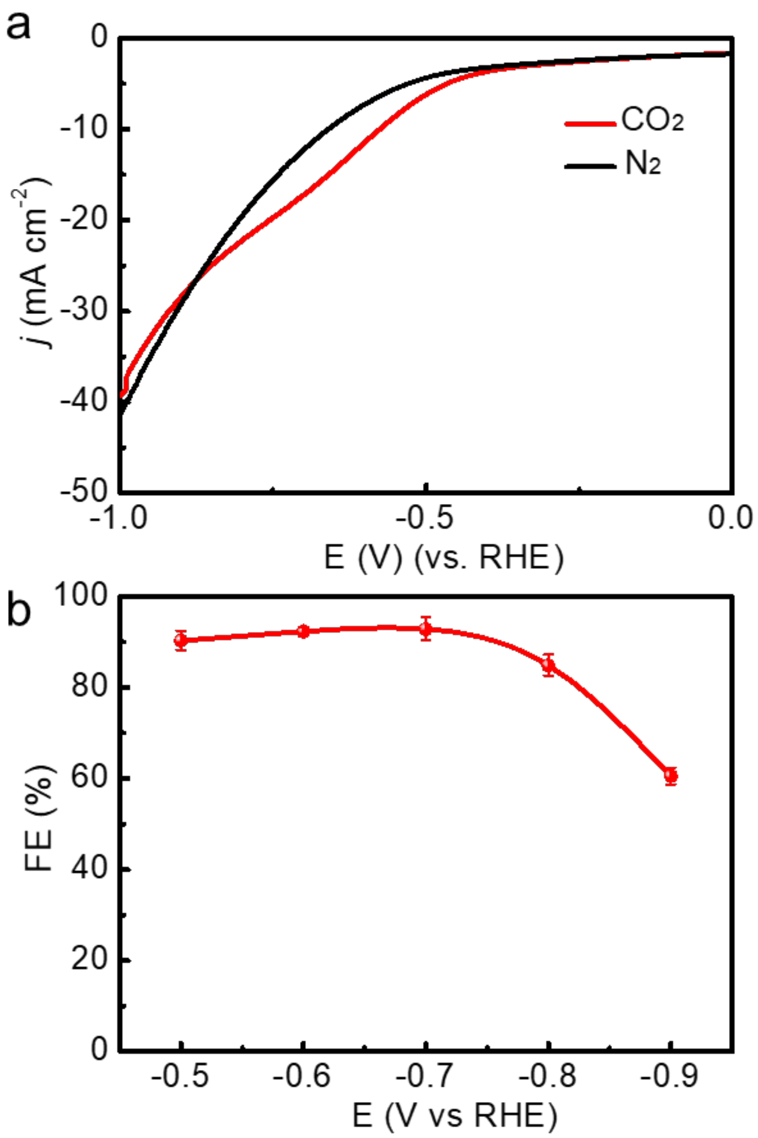


**Supplementary Figure 33 | Electrochemical performance of (Br, N)-Mn/G electrocatalyst.** LSV curves (a) and CO faradaic efficiencies (b) of (Br, N)-Mn/G at various applied potentials.

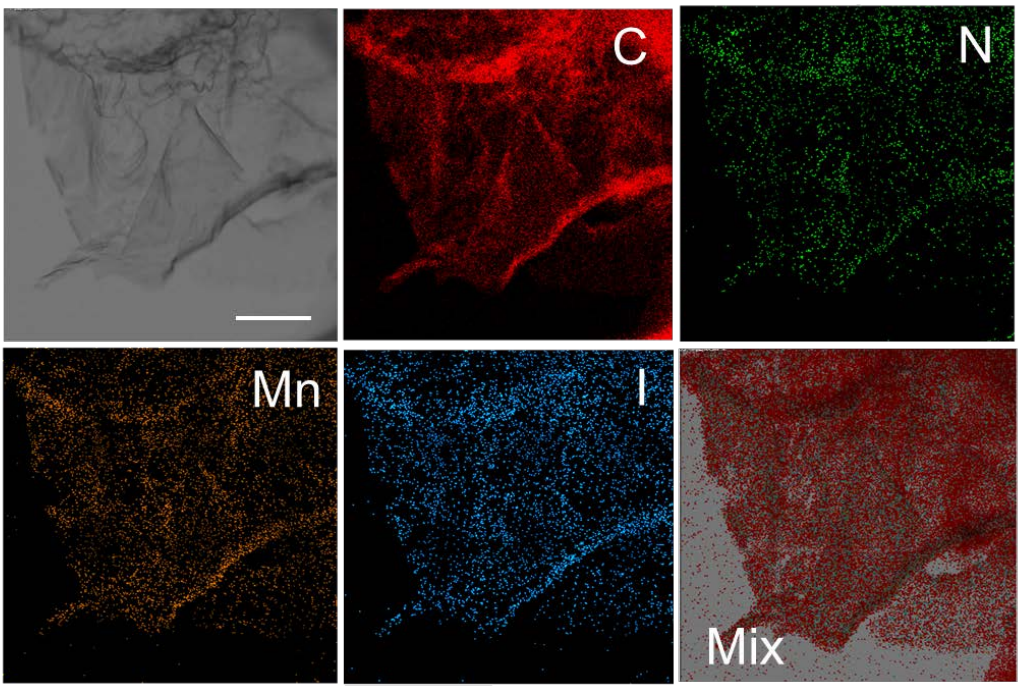


**Supplementary Figure 34 | Structural characterizations of (I, N)-Mn/G.** TEM image and corresponding EDS images of (I, N)-Mn/G. Scale bar, 500 nm.

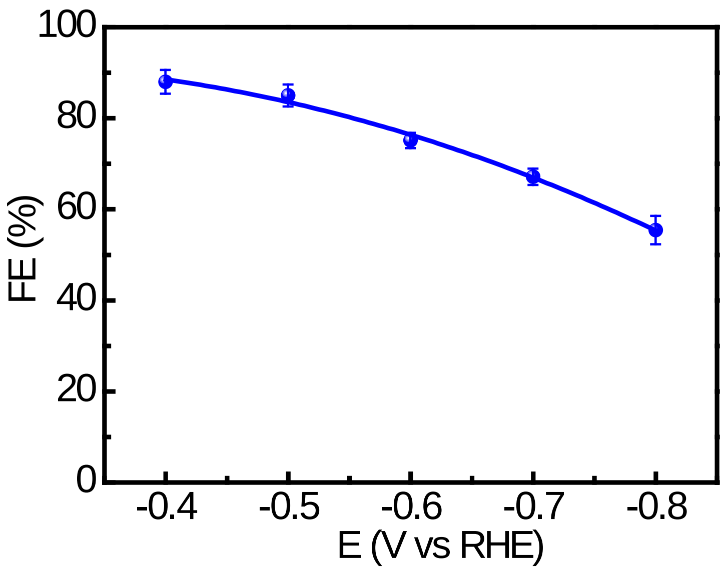


**Supplementary Figure 35 | Electrochemical performance of (I, N)-Mn/G electrocatalyst.** CO faradaic efficiencies of (I, N)-Mn/G at various applied potentials.

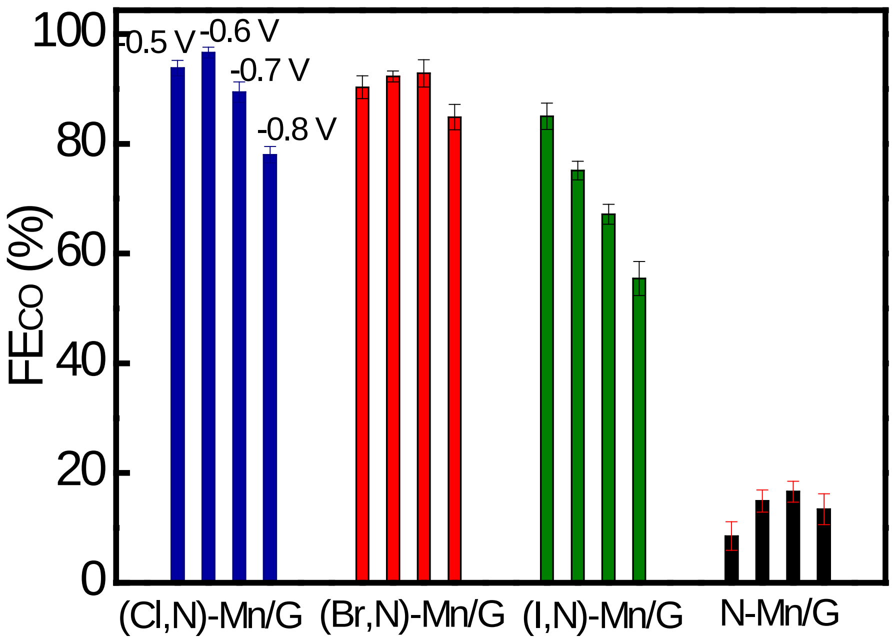


**Supplementary Figure 36 | Comparison of the electrochemical performance for dual-coordinated Mn electrocatalysts.** CO faradaic efficiencies of (Cl, N)-Mn/G, (Br, N)-Mn/G, (I, N)-Mn/G and N-Mn/G at various applied potentials.

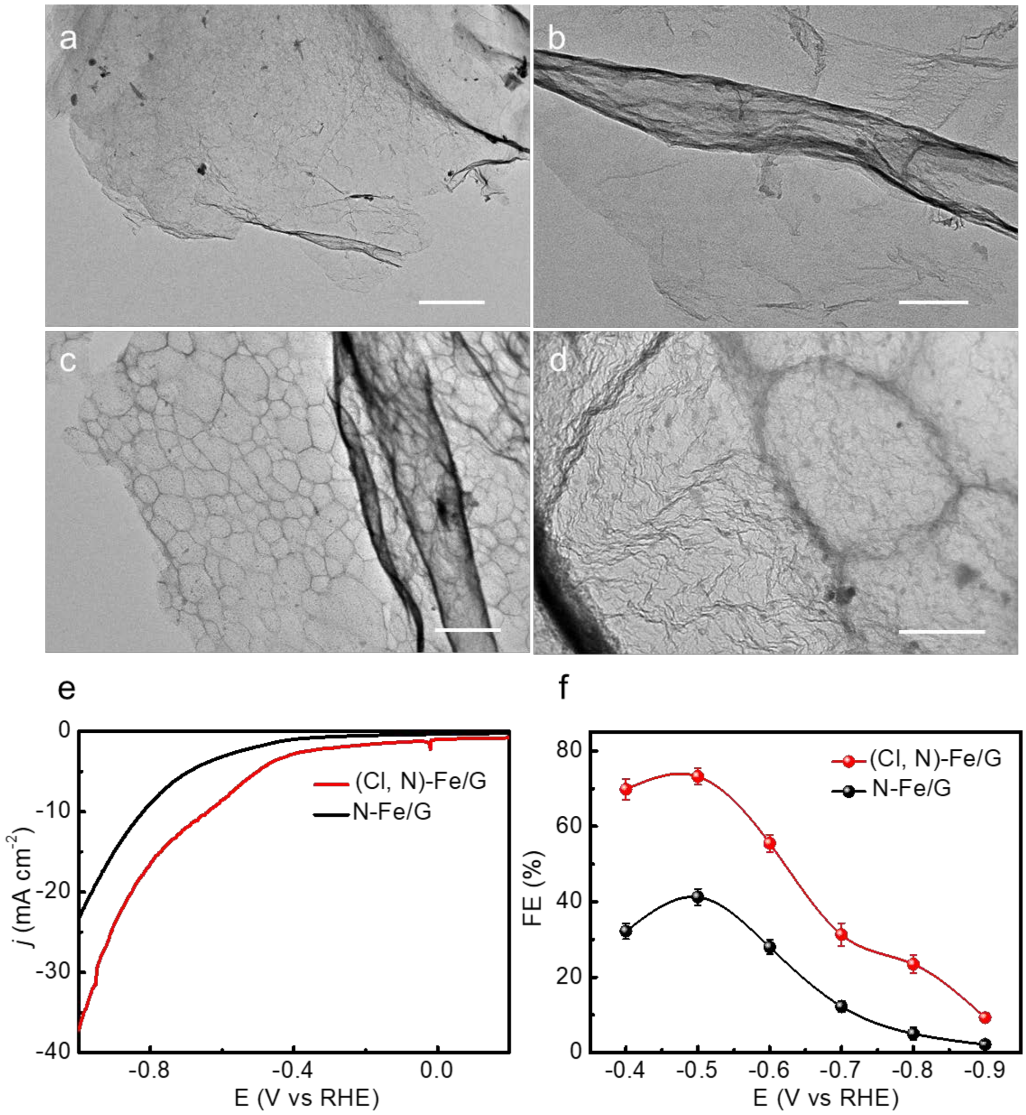


**Supplementary Figure 37 | Characterizations and electrochemical $CO_2$RR performance of (Cl, N)-Fe/G.** TEM images of (Cl, N)-Fe/G (a, b) and N-Fe/G (c, d). Corresponding LSV curves (e) and CO faradaic efficiencies (f) at various applied potentials. Scale bar, 500 nm in (a), (c) and 100 nm in (b), (d).

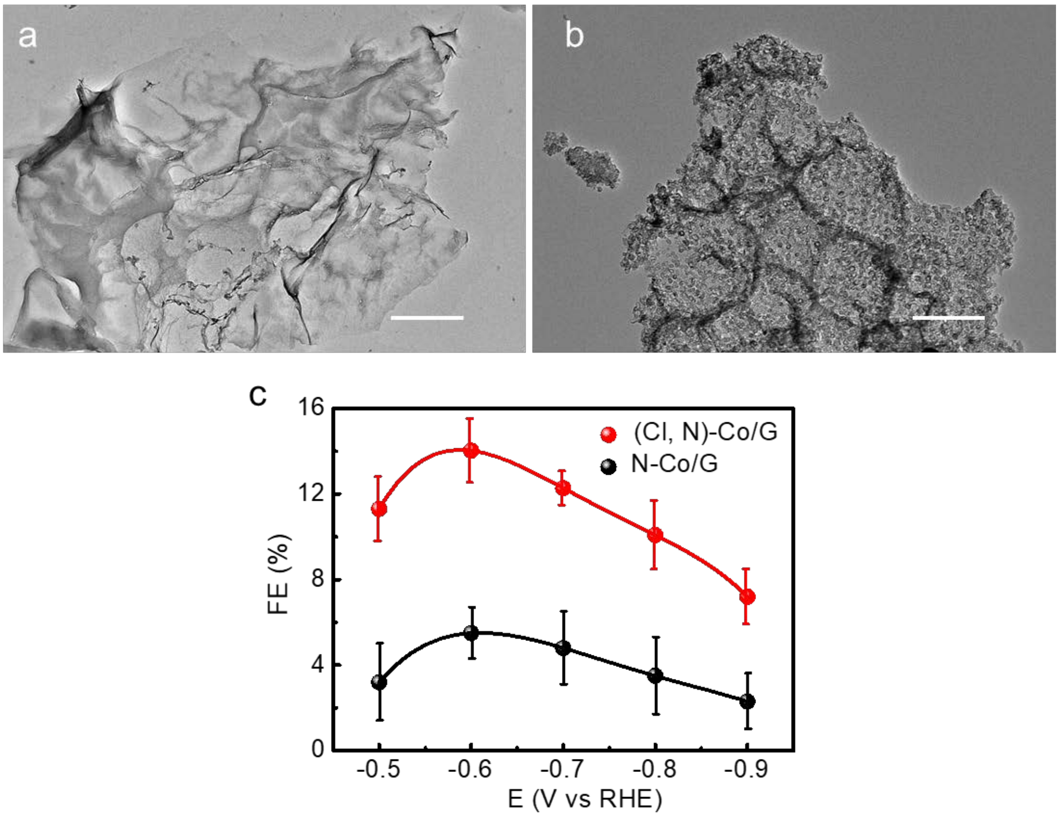


**Supplementary Figure 38 | Characterizations and electrochemical $CO_2$RR performance of (Cl, N)-Co/G.** TEM images of (Cl, N)-Co/G (a) and N-Co/G (b) and corresponding CO faradaic efficiencies at various applied potentials (c). Scale bar, 500 nm in (a) and 200 nm in (c).

**Supplementary Table 1 |** Chemical compositions of (Cl, N)-Mn/G and N-Mn/G by elemental analysis[a] and ICP measurement[b].

| Sample | C (wt%)[a] | N (wt%)[a] | Cl (wt%)[a] | Mn (wt%)[b] |
|---|---|---|---|---|
| (Cl, N)-Mn/G | 82.8 | 4.2 | 2.1 | 0.049 |
| N-Mn/G | 85.4 | 3.9 | – | 0.14 |

**Supplementary Table 2 |** Best fitting EXAFS data for different Mn-based catalysts.

| Sample | Scattering Pair | *CN* | *R* (Å) | $\sigma^2$ ($Å^2$) | $\Delta E_0$ (eV) | *R* factor |
|---|---|---|---|---|---|---|
| (Cl, N)-Mn/G | Mn-N | 3.7 | 2.08 | 0.00903 | -9.5 | 0.014 |
| | Mn-Cl | 1.4 | 2.39 | 0.01340 | 4.3 | |
| N-Mn/G | Mn-N | 3.9 | 2.02 | 0.02981 | -2.1 | 0.01586 |
| Mn(II)Pc | Mn-N | 4 | 1.95 | 0.00047 | 2.1 | 0.00560 |

*CN* is the coordination number; *R* is interatomic distance (the bond length between central atoms and surrounding coordination atoms); $\sigma^2$ is Debye-Waller factor (a measure of thermal and static disorder in absorber-scatterer distances); $\Delta E_0$ is edge-energy shift (the difference between the zero kinetic energy value of the sample and that of the theoretical model). *R* factor is used to value the goodness of the fitting. Error bounds that characterize the structural parameters obtained by EXAFS spectroscopy were estimated as N ± 20%; R ± 1%; $\sigma^2$ ± 20%; $\Delta E_0$ ± 20%.

**Supplementary Table 3 |** DFT total energies ($E_{\mathrm{DFT}}$), zero-point energies ($E_{\mathrm{ZPE}}$), entropies ($T*S$) multiplied by temperature ($T$ = 300 K), free energies ($G$), relative free energies ($\Delta G$) at $U$ = 0 versus SHE, and adsorption energies of $CO_2$ reduction reaction intermediates on (Cl, N)-Mn/G and N-Mn/G.

| Catalysts | $E_{\mathrm{DFT}}$ (eV) | $E_{\mathrm{ZPE}}$ (eV) | $T*S$ (eV) | $G$ (eV) | $\Delta G$ (eV) | $E_{\mathrm{ads}}$ (eV) |
|---|---|---|---|---|---|---|
| (Cl, N)-Mn/G | -118190.668 | 11.860 | 1.479 | -118180.307 | -- | -- |
| COOH* | -123335.702 | 12.484 | 1.684 | -123324.902 | -0.69 | -2.36 |
| CO* | -121272.808 | 12.223 | 1.532 | -121262.117 | -0.60 | -0.91 |
| H* | -118205.867 | 12.142 | 1.481 | -118195.206 | 0.92 | 0.64 |
| N-Mn/G | -105671.659 | 11.789 | 1.394 | -105661.264 | -- | -- |
| COOH* | -110815.935 | 12.453 | 1.610 | -110805.092 | 0.29 | -1.62 |
| CO* | -108754.789 | 12.158 | 1.453 | -108744.084 | -1.59 | -1.90 |
| H* | -105687.667 | 12.071 | 1.396 | -105676.992 | 0.11 | -0.17 |

**Supplementary Table 4 |** Geometry configuration parameters about (Cl, N)-Mn/G, N-Mn/G and intermediate species.

| Catalysts | | Bond length (Å) | | | $E_{dbc}$ (eV) |
|---|---|---|---|---|---|
| | | M-N | M-C | Mn-Cl | |
| (Cl, N)-Mn/G | * | 1.941 | -- | 2.173 | -1.228 |
| | COOH* | 1.927 | 2.086 | 2.307 | -1.606 |
| N-Mn/G | * | 1.908 | -- | -- | -1.027 |
| | COOH* | 1.923 | 1.962 | -- | -1.146 |

**Supplementary Table 5 |** Performance comparison of various reported $CO_2$RR electrocatalysts.[a]

| Catalysts | Cathode Loading (mg cm$^{-2}$) | $FE_{CO}$ | $j_{CO}$ (mA·cm$^{-2}$) | Potential (V) vs. RHE | TOF (h$^{-1}$) | Ref. |
|---|---|---|---|---|---|---|
| **(Cl, N)-Mn/G** | 0.5 | 97% | 9.2 | -0.6 | 38347 | This work |
| **(Cl, N)-Mn/G** | 0.5 | 94% | 4.0 | -0.5 | 25008 | This work |
| **(Cl, N)-Mn/G** | 0.5 | 90% | 12.3 | -0.7 | 51268 | This work |
| **Mn-N-C** | 0.76 | ~40% | ~0.8 | -0.55 | - | 14 |
| **Mn-N-C** | 0.79 | ~65% | ~3.3 | -0.6 | - | 15 |
| **Mn-N-C** | 0.3 | ~70% | 0.7~ | -0.6 | - | 16 |
| **Co-N-C** | 0.76 | ~20% | 1.2 | -0.5 | - | 14 |
| **$ZnN_x$/C** | 1 | 95% | 1~ | -0.43 | 1416 | 17 |
| | 1 | ~85% | ~6 | -0.63 | 8390 | |
| **Fe-$N_4$** | 1 | 80% | ~2.7 | -0.6 | - | 18 |
| **COF-367-Co** | - | 91% | 3.3 | -0.67 | 1908 | 19 |
| **CoPc/CNT** | 0.4 | 92% | ~10 | -0.63 | - | 20 |
| **CoPPc/CNT** | 1 | ~90% | ~8 | -0.5 | - | 21 |
| **Co-$N_2$** | 0.8 | 94% | 17 | -0.63 | 18200 | 22 |
| **Co-$N_5$/HNPCSs** | - | 99.3% | 10.1 | -0.79 | - | 23 |
| | - | ~94% | ~2.5 | -0.6 | - | |
| **CoPc-P4VP** | - | 89% | 1.78 | -0.73 | - | 24 |
| **Ni SAs/N-C** | 0.1 | 71.9% | 7.5 | -1 | 5273 | 25 |
| **A-Ni-NSG** | 0.4 | 94% | 22.1 | -0.72 | 2960 | 1 |
| | 0.4 | - | ~9 | -0.6 | - | |
| **Ni-$N_4$-C** | 0.2(GC)[b] | 99% | ~15 | -0.81 | - | 6 |
| | 0.2(GC)[a] | 98% | ~9 | -0.6 | - | |

| | | | | | | |
|---|---|---|---|---|---|---|
| **SE-Ni SAs@PNC** | 0.4 | 95% | ~2 | -0.6 | 4006 | 26 |
| | 0.4 | 88% | 18.3 | -1 | 47805 | |
| **N-based silver** | 0.1 | 90% | 90 | -1.6 | 2000 | 27 |
| **Nanoporous silver** | - | 92% | 8.7 | -0.5 | 7.2 | 9 |
| **Pd nanoparticles** | 2.0 | 91% | 9.76 | -0.89 | 576 | 12 |
| **Ultrathin Au NWs** | - | 94% | 8.16 | -0.35 | 72 | 13 |

[a] 0.5 M $KHCO_3$ was used as electrolyte for all the $CO_2$ RR listed in the table except Ref. 13 and 27 (0.1 M $KHCO_3$). [b]Calculated on the Glassy Carbon Electrode (GC).

## Supplementary references


1. Yang, H. B. et al. Atomically dispersed Ni(I) as the active site for electrochemical $CO_2$ reduction. *Nat. Energy* **3**, 140-147 (2018).
2. Domenick F. L. & Timothy A. Jackson. Mn K-edge X-ray absorption studies of oxo- and hydroxo-manganese(IV) complexes: Experimental and theoretical insights into pre-edge properties. *Inorg. Chem*. **53**, 6179-6194, (2014).
3. Fei, H. L. et al. General synthesis and definitive structural identification of $MN_4C_4$ single-atom catalysts with tunable electrocatalytic activities. *Nat. Catal*. **1**, 63-72 (2018).
4. Li, Q. H. et al. Fe Isolated single atoms on S, N codoped carbon by copolymer pyrolysis strategy for highly efficient oxygen reduction reaction. *Adv. Mater.* **30**, 1800588 (2018).
5. Liu, W. et al. Single-site active cobalt-based photocatalyst with long carriers lifetime for spontaneous overall water splitting. *Angew. Chem. Int. Ed*. **56**, 9312-9317 (2017).
6. Li, X. G. et al. Exclusive Ni-$N_4$ sites realize near-unity CO selectivity for electrochemical $CO_2$ reduction. *J. Am. Chem. Soc*. **139**, 14889-14892 (2017).
7. Liu, M. et al. Enhanced electrocatalytic $CO_2$ reduction via field-induced reagent concentration. *Nature* **537**, 382-386 (2016).
8. Rogers, C. et al. Synergistic enhancement of electrocatalytic $CO_2$ reduction with gold nanoparticles embedded in functional graphene nanoribbon composite electrodes. *J. Am. Chem. Soc.* **139**, 4052-4061 (2017).
9. Lu, Q. et al. A selective and efficient electrocatalyst for carbon dioxide reduction. *Nat. Commun*. **5**, 3242 (2014).
10. Kim, C. *et al*. Achieving selective and efficient electrocatalytic activity for $CO_2$ reduction using immobilized silver nanoparticles. *J. Am. Chem. Soc*. **137**, 13844-13850 (2015).
11. Ma, M., Trzesniewski, B. J., Xie, J. & Smith, W. A. Selective and efficient

reduction of carbon dioxide to carbon monoxide on oxide-derived nanostructured silver electrocatalysts. *Angew. Chem., Int. Ed.* **55**, 9748-9752 (2016).

12. Gao, D. F. et al. Size-Dependent electrocatalytic reduction of $CO_2$ over Pd nanoparticles. *J. Am. Chem. Soc*. **137**, 4288-4291 (2015).
13. Zhu, W. *et al*. Active and selective conversion of $CO_2$ to CO on ultrathin Au nanowires. *J. Am. Chem. Soc*. **136**, 16132-16135 (2014).
14. Ju, W. et al. Understanding activity and selectivity of metal-nitrogen-doped carbon catalysts for electrochemical reduction of $CO_2$. *Nat. Commun.* **8**, 944 (2017).
15. Varela, A. S. et al. Metal-doped nitrogenated carbon as an efficient catalyst for direct $CO_2$ electroreduction to CO and hydrocarbons. *Angew. Chem. Int. Ed*. **54**, 10758-10762 (2015).
16. Pan, F. P., Deng, W., Justiniano, C & Li, Y. Identification of champion transition metals centers in metal and nitrogencodoped carbon catalysts for $CO_2$ reduction. *Appl. Catal. B: Environ*. **226**, 463-472 (2018).
17. Yang, F. et al. Highly efficient $CO_2$ electroreduction on $ZnN_4$-based single-atom catalyst. *Angew.Chem. Int. Ed*. **57**, 12303-12307 (2018).
18. Zhang, C. H. et al. Electrochemical $CO_2$ reduction with atomic iron-dispersed on nitrogen-doped graphene. *Adv. Energy Mater*. **8**, 1703487 (2018).
19. Lin, S. et al. Covalent organic frameworks comprising cobalt porphyrins for catalytic $CO_2$ reduction in water. *Science* **349**, 1208-1213 (2015).
20. Zhang, X. et al. Highly selective and active $CO_2$ reduction electro-catalysts based on cobalt phthalocyanine/carbon nanotube hybrid structures. *Nat. Commun*. **8**, 14675 (2017).
21. Han, N. et al. Supported cobalt polyphthalocyanine for high-performance electrocatalytic $CO_2$ reduction. *Chem* **3**, 652-664 (2017).
22. Wang, X. Q. et al. Regulation of coordination number over single Co sites: Triggering the efficient electroreduction of $CO_2$. *Angew. Chem. Int. Ed*. **57**, 1944-1948 (2018).
23. Pan, Y. et al. Design of single-atom Co-$N_5$ catalytic site: A robust electrocatalyst for $CO_2$ reduction with nearly 100% CO selectivity and remarkable stability. *J.*

*Am. Chem. Soc*. **140**, 4218-4221 (2018).

24. Kramer, W. W. & McCrory C. C. L. Polymer coordination promotes selective $CO_2$ reduction by cobalt phthalocyanine. *Chem. Sci*. **7**, 2506-2515 (2016).

25. Zhao, C. M. et al. Ionic exchange of metal-organic frameworks to access single nickel sites for efficient electroreduction of $CO_2$. *J. Am. Chem. Soc.* **139**, 8078-8081 (2017).

26. Yang, J. et al. In-situ thermal atomization to transfer supported metal nanoparticles to surface enriched Ni single atom catalyst. *Angew. Chem. Int. Ed*. **57**, 14095-14100 (2018).

27. Tornow, C. E., Thorson, M. R., Ma, S., Gewirth, A. A & Kenis, P. J. A. Nitrogen-based catalysts for the electrochemical reduction of $CO_2$ to CO. *J. Am. Chem. Soc*. **134**, 19520-19523 (2012).
