# Supplementary material for: Manganese acting as a high-performance heterogeneous electrocatalyst in carbon dioxide reduction
Source: Nat Commun. 2019 Jul 5;10:2980. doi: 10.1038/s41467-019-10854-1 (PMC6611886; doi:10.1038/s41467-019-10854-1)
Supplement: Supplementary file 2 — Reporting Summary [file 41467_2019_10854_MOESM2_ESM.pdf]

nature research

Corresponding author(s): Jianling Zhang

Last updated by author(s): May 13, 2019

Reporting Summary

Nature Research wishes to improve the reproducibility of the work that we publish. This form provides structure for consistency and transparency in reporting. For further information on Nature Research policies, see Authors & Referees and the Editorial Policy Checklist .

Statistics

For all statistical analyses, confirm that the following items are present in the figure legend, table legend, main text, or Methods section.

n/a	Confirmed	
☐	☒	The exact sample size (*n*) for each experimental group/condition, given as a discrete number and unit of measurement
☐	☒	A statement on whether measurements were taken from distinct samples or whether the same sample was measured repeatedly
☐	☒	The statistical test(s) used AND whether they are one- or two-sided<br>*Only common tests should be described solely by name; describe more complex techniques in the Methods section.*
☒	☐	A description of all covariates tested
☐	☒	A description of any assumptions or corrections, such as tests of normality and adjustment for multiple comparisons
☐	☒	A full description of the statistical parameters including central tendency (e.g. means) or other basic estimates (e.g. regression coefficient) AND variation (e.g. standard deviation) or associated estimates of uncertainty (e.g. confidence intervals)
☒	☐	For null hypothesis testing, the test statistic (e.g. *F*, *t*, *r*) with confidence intervals, effect sizes, degrees of freedom and *P* value noted<br>*Give P values as exact values whenever suitable.*
☒	☐	For Bayesian analysis, information on the choice of priors and Markov chain Monte Carlo settings
☒	☐	For hierarchical and complex designs, identification of the appropriate level for tests and full reporting of outcomes
☒	☐	Estimates of effect sizes (e.g. Cohen's *d*, Pearson's *r*), indicating how they were calculated

Our web collection on statistics for biologists contains articles on many of the points above.

Software and code

Policy information about availability of computer code

Data collection	The apparatus and software used for data collection are provided in detail in Method Section of the main text and Supplementary Information, and necessary references are also provided.
Data analysis	The method and software used for data analysis and calculation are provided in detail in Method Section of the main text and Supplementary Information, and necessary references are also provided. All relevant data are available from the authors.

For manuscripts utilizing custom algorithms or software that are central to the research but not yet described in published literature, software must be made available to editors/reviewers. We strongly encourage code deposition in a community repository (e.g. GitHub). See the Nature Research guidelines for submitting code & software for further information.

Data

Policy information about availability of data

All manuscripts must include a data availability statement. This statement should provide the following information, where applicable:

- Accession codes, unique identifiers, or web links for publicly available datasets
- A list of figures that have associated raw data
- A description of any restrictions on data availability

The data supporting the findings of this study are available within the article and its Supplementary information files. All data is available from the authors upon reasonable request.

Field-specific reporting

Please select the one below that is the best fit for your research. If you are not sure, read the appropriate sections before making your selection.

Ecological, evolutionary & environmental sciences study design

All studies must disclose on these points even when the disclosure is negative.

Study description	The brief description is given in the last paragraph of Introduction.
Research sample	No biological sample was used for the research.
Sampling strategy	The thickness of the sample was obtained by the AFM measurements.
Data collection	The data collection methods are described in Method Section of the main text and Supplementary Information. The data were recorded by Bingxing Zhang and Jianling Zhang.
Timing and spatial scale	All the data were obtained from Mar., 2018 to Nov., 2018.
Data exclusions	No data were excluded from the analyses.
Reproducibility	The methods of reproduced the experiment are described in Method Section of the main text and Supplementary Information.For Faradaic efficiency data, the experiment at each condition was repeated three times.
Randomization	The samples were allocated by molar ratio of the metal which was used in preparation of the materials.
Blinding	Blinding was not applied.

Did the study involve field work? ☐ Yes ☒ No

Reporting for specific materials, systems and methods

We require information from authors about some types of materials, experimental systems and methods used in many studies. Here, indicate whether each material, system or method listed is relevant to your study. If you are not sure if a list item applies to your research, read the appropriate section before selecting a response.

Materials & experimental systems

n/a	Involved in the study
☒	☐ Antibodies
☒	☐ Eukaryotic cell lines
☒	☐ Palaeontology
☒	☐ Animals and other organisms
☒	☐ Human research participants
☒	☐ Clinical data

Methods

n/a	Involved in the study
☒	☐ ChIP-seq
☒	☐ Flow cytometry
☒	☐ MRI-based neuroimaging
